# Molecular classification of blood and bleeding disorder genes

**DOI:** 10.1038/s41525-021-00228-2

**Published:** 2021-07-16

**Authors:** Batoul Baz, Mohamed Abouelhoda, Tarek Owaidah, Majed Dasouki, Dorota Monies, Nada Al Tassan

**Affiliations:** 1 Saudi Human Genome Program, National Centre for Genomic Technologies, King Abdulaziz City for Science and Technology, Riyadh, Saudi Arabia; 2 Department of Genetics, Research Centre, King Faisal Specialist Hospital and Research Centre, Riyadh, Saudi Arabia; 3 Systems and Biomedical Engineering Department, Faculty of Engineering, Cairo University, Giza, Egypt; 4 Department of Pathology & Laboratory Medicine, King Faisal Specialist Hospital and Research Centre, Riyadh, Saudi Arabia

**Keywords:** Disease genetics, Genetics research

## Abstract

The advances and development of sequencing techniques and data analysis resulted in a pool of informative genetic data, that can be analyzed for informing decision making in designing national screening, prevention programs, and molecular diagnostic tests. The accumulation of molecular data from different populations widen the scope of utilization of this information. Bleeding disorders are a heterogeneous group of clinically overlapping disorders. We analyzed the targeted sequencing data from ~1285 Saudi individuals in 17 blood and bleeding disorders genes, to determine the frequency of mutations and variants. We used a replication set of ~5000 local exomes to validate pathogenicity and determine allele frequencies. We identified a total of 821 variants, of these 98 were listed in HGMD as disease related variants and 140 were novel variants. The majority of variants were present in *VWF*, followed by *F5*, *F8*, and *G6PD* genes, while *FGG*, *FGB*, and *HBA1* had the lowest number of variants. Our analysis generated a priority list of genes, mutations and novel variants. This data will have an impact on informing decisions for screening and prevention programs and in management of vulnerable patients admitted to emergency, surgery, or interventions with bleeding side effects.

## Introduction

Clinical molecular laboratories have focused on providing molecular testing for single gene disorders, where the phenotype can be explained by a specific mutation in a single gene^1^. Using Sanger sequencing; identifying causative mutations was the primary molecular diagnostic tool, where most mutations were recurrent and easily identified^2^. Molecular diagnostic tests for single gene disorders have undergone great technological advances, allowing diagnostic laboratories to expand the list of genes tested^3^. In addition to identifying point mutations, more complicated genetic events were also included in routine diagnostic tests (e.g., Fragile X testing^4^, Down syndrome^5^). As the list of causative genes and variants in both monogenic and complex disorders is enriched through massive research efforts, molecular testing has demanded advanced techniques beyond Sanger sequencing to cover more target regions. For example, more than 2000 sequence variations have been reported in the *CFTR* gene, and the majority of these have to be considered in diagnostic testing and reporting^2^.

Targeted Next Generation Sequencing (NGS) of a disease-specific subset of genes has shown to be efficient and of the same level of accuracy as Sanger sequencing, and hence has been reliably implemented as a stand-alone cost effective diagnostic test^6–8^. These techniques allowed high throughput screening of multiple genes or whole exomes to identify disease causative mutations. Research efforts using NGS has allowed researchers to characterize the molecular basis of different monogenic and multigene disorders^9^. Also, it contributed to disease and population-specific single nucleotide variants (SNVs) databases, such as; ClinVar, the Human Genetic Variation Database (HGVD), and HbVar database of human hemoglobin variants and thalassemia mutations^9–13^. These databases are very important in replication studies and for classification of identified variants. Introducing these technologies in the clinical diagnostic filed is the first step towards the era of personalized medicine.

Around the world, a number of large-scale initiatives are poised to bring NGS-based tests into routine medical care. Implementation of such programs is expanding globally using different approaches; population genomic sequencing and genomic research programs that are sometimes combined with detailed phenotyping and links to electronic medical records, and inclusion of ethnic specific genetic data that highlight population differences^14^. Patient registries allow informed worldwide health policy decisions and there are different national and international rare and complex disease registries and consortiums that combine clinical, genetic data, and bio banking in some cases^15–17^. The optimal analysis of the big NGS data aims at extracting information and make this information available to be translated into knowledge and action^18^. To achieve this, automated high-performance computing is needed. Analysis of big clinical and genetic data in health can be used to improve the efficacy of prediction and prevention policies of medical interventions and treatments, health delivery services, and most importantly general health policies and visions. This will ultimately improve outcomes for individual patients through earlier diagnosis, better treatment plans and prognosis, and improved decision support for clinicians. In Saudi Arabia, blood disorders registries efforts so far are limited and research is focused on mutational screening papers, however, on the other hand, genomics is well established with a solid foundation to enable the practice of precision medicine.

The Saudi Human Genome Program (SHGP) is a national Saudi Arabian initiative aiming to sequence 100,000 genomes. In its first phase, it directed its efforts on molecular investigation of monogenic disorders. Then expanded to understand the genetics of complex disorders and the molecular landscape of this highly inbred population. The data generated from of this program was used to establish a repository of population specific mutations and variants that can be used for molecular diagnosis and profiling. Targeted sequencing at the SHGP resulted in molecular diagnosis in nearly half of ~2500 cases with Mendelian monogenic disorders using 13 gene panels^17^. To date; the SHGP repository has referenced and validated more than 2000 disease causing mutations and documented than 4 million SNVs.

Hemostasis is a dynamic process that involves a cascade of coagulation proteins that aggregate and rapidly form a clot. The process involves the production of thrombin, which subsequently activates other coagulation factors in order to create stable blood clots. Such a complicated process is controlled by various genetic factors. With many players involved in different bleeding disorders, it is crucial to characterize the phenotype genotype correlation in order to provide the best healthcare management plan. To establish the diagnosis of the rare bleeding disorders, physicians depend mainly on different coagulation tests, which has some limitations and can be affected by many factors resulting in delayed or incorrect diagnosis. Molecular diagnosis is the concrete and most reliable in some cases of blood and bleeding disorders, where other tests do not provide conclusive evidence of a pathological condition^19^. Establishing a conclusive molecular diagnosis helps in providing the best patient care in terms of therapy, management, prognosis, and counseling.

In blood and bleeding disorders, examples of targeted gene panels are the SHGP heme^20^ and the ThromboGenomics consortium panels^21^. In the current study, we analyzed the data from the SHGP to redefine and classify both pathogenic and benign variants in 17 selected genes that are associated with different blood and bleeding disorders. First, we evaluated a training set (P-cohort) of 1285 individuals using a targeted gene panel consisting of 393 genes implicated in nonmalignant blood related disorders (SHGP heme panel). We designed our filtering process to identify novel variants of interest and previously reported mutations in bleeding disorders based on the incidence of carriers and affected individuals. We used replication set (R-cohort) of ~5000 whole exome sequencing (WES) to validate the variants identified in the training set and estimate the population allele frequencies. This data will impact decision making for designing effective screening and prevention programs, and management plans for patients susceptible to bleeding disorders. This unlimited approach of mining population data will aid in improving the diagnostic yield, and identifying population specific molecular markers and in developing strategies to manage susceptible patients/cases, where unexplained suspected bleeding might occur.

## Results

### NGS yield and variants

In total, 1285 cases in the P-cohort have been sequenced and passed the QC criteria. Each sample had an average depth of 250× and the target regions covered at least 95% at 10×. Average QC values of the sequenced samples are summarized in the (Supplementary Table 1).

Each variant is reported only if it is covered by at least 100 reads and its quality score is more than 50. On average, each variant file for the heme panel covering the 393 genes included 1300 variants. After filtration, the number dropped to 18 variants. Further filtering based on the selected genes of interest retained an average of four variants per file. The total yield (presence of likely causative variant) is 38.36%, where 493 out 1285 cases harbored a pathogenic or likely pathogenic variant in the genes of interest. In general, *VWF*, *F5*, *F8*, and *G6PD* reported the highest number of variants, while *FGG*, *FGB*, and *HBA1* accumelated the lowest number of variants (Supplementary Table 2 and Supplementary Fig. 1).

### Previously reported pathogenic variants

**Fig. 1:**
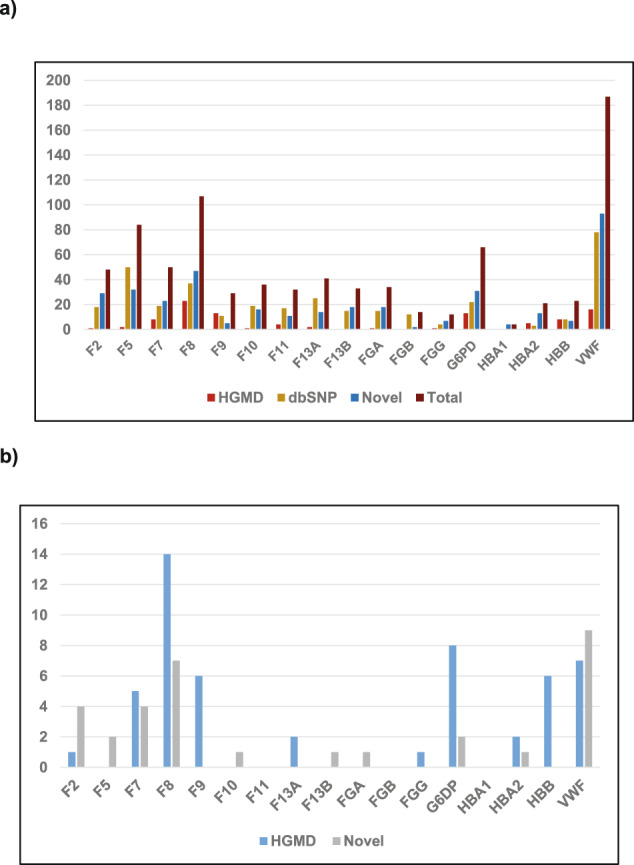
Count and classification of variants identified in P-Cohort. **a** Count of HGMD, Novel variants and polymorphisms/ gene (generated from Supplementary Table 2). **b** Number of Pathogenic and likely pathogenic HGMD and novel mutations. (generated from Supplementary Tables 3 and 4).

We identified 98 variants that were listed in HGMD; 12 truncating, 11 splice and UTR, one stop loss, 2 synonymous and 72 nonsynonymous. Of those 52 were re-classified as pathogenic, likely pathogenic, 19 as variants of unknown significance (VUS) and 27 as benign or likely benign (Supplementary Tables 2 and 3 and Supplementary Fig. 1). Fifty six of these had a CADD score >20. *F8*, *VWF*, *G6PD*, *F9*, and *F7* recorded the highest number of previously reported variants (Fig. 1a). *F8* has the highest pathogenic/likely pathogenic variants followed by *G6PD*, *VWF*, *F9*, and *HBB*. Interestingly, no HGMD variants were recorded in *F13B*, *FGB*, and *HBA1* after filtering (Fig. 1b and Supplementary Table 3). Only 430 cases in our P-cohort harbored a pathogenic or likely pathogenic previously reported variant.

### Novel variants

One hundred and forty variants were identified; 4 truncating, 13 frameshifts, 6 splice, and 117 nonsynonymous. Seventy-one of these reported variants had a CADD score above 20. Thirty-two variants were classified as pathogenic or likely pathogenic, 105 were classified as VUS and only three were ranked as likely benign (Supplementary Tables 2 and 4 and Supplementary Fig. 1). Over all, *VWF* had the highest novel variants followed by *F8*, *F5*, *G6PD*, and *F2* (Fig. 1a), while *F8* and *VWF* recorded the highest number of pathogenic and likely pathogenic classified mutations. No variants in *F11*, *FGB*, and *HBA1* were retained by our filtering process (Fig. 1b, and Supplementary Table 4). In our P-cohort 63 cases carried a pathogenic or likely pathogenic novel variant. It is worth noting that 3 of the 4 novel truncating variants were identified in two cases of aplastic anemia, and a case of excessive bleeding of which the main causative variant was not identified.

### Polymorphisms

Our filtering retained 354 SNPs that are listed in dbSNP; 2 truncating, 98 nonsynonymous, 96 synonymous and 158 intronic and UTR changes. *VWF*, *F5*, and *F8* had the highest number of SNPs (Supplementary Tables 2 and 5, Fig. 1a, and Supplementary Fig. 1).

### Carrier frequency (CF) in autosomal recessive (AR) genes

**Fig. 2:**
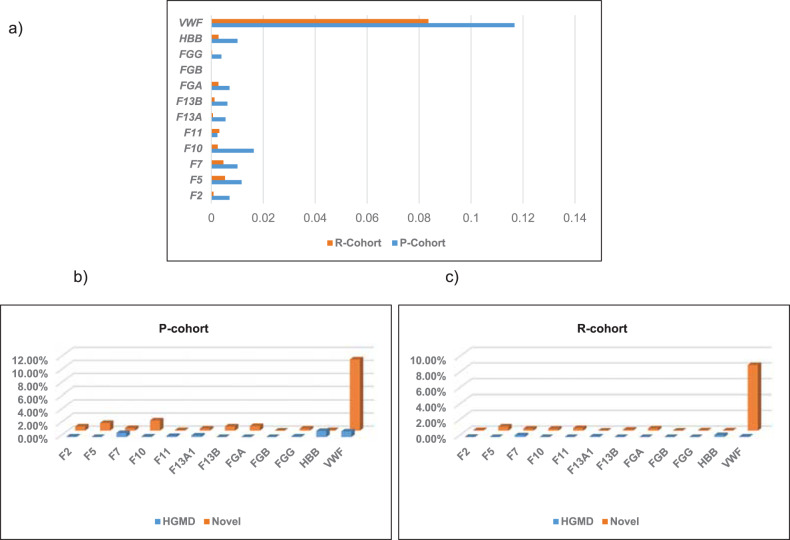
Heterozygotes frequency in AR genes. **a** Carrier status for pathogenic, likely pathogenic and VUS/gene in both cohorts. **b** Percentage of HGMD and novel mutations carriers/gene for P-cohort. **c** Percentage of HGMD and novel mutations carriers/gene for R-cohort. (generated from Supplementary Tables 6 and 7).

We calculated the CF for each HGMD and novel variants in both cohorts (Supplementary Tables 3 and 4). We also calculated the overall CF for the 12 AR genes (Supplementary Tables 6 and 7 and Fig. 2). As expected *WVF* had the highest CF (0.116 and 0.0835) in P and R cohorts respectively, followed by *F10*, *HBB*, *F7*, and *F5*. Interestingly, there were no carriers detected in *FGG* in our cohorts.

## Discussion

Genetic data plays a confirmatory role in clinical assessment and diagnosis. The development of high throughput sequencing techniques and the availability of normal population variants and patient mutation databases helps in translating genetic data into an important sensitive diagnostic tool. That, in combination with clinical observations and other forms of investigation, can proactively help in formulating clinical decisions and patients management plans^6^. The availability of different commercial gene panels and WES services is an indication that genetic data acquisition and interpretation is no longer a challenge, and can be used not just as a reliable diagnostic tool for inherited disorders but also as a health management tool for complex disorders such as cancer^1^.

The majority of bleeding disorders are inherited as autosomal recessive (AR) but heterozygous carriers might present with variable degrees of the correlated factor deficiency, which may result in an increased tendency for bleeding^22^. In patients with defined bleeding symptoms, hematological laboratory profiling combined with molecular techniques result in accurate diagnosis and is the foundation for the required genetic counseling, especially in populations with high rates of consanguinity. In some highly inbred populations, consanguineous marriages may increase the burden of mutations in genes associated with AR diseases^23^. Bleeding may be considered as a risk factor for patients undergoing major surgeries, dialysis, and specific treatments where a hidden underlying genetic susceptibility should be investigated and considered^24,25^. Putting all this information in the context of improvement of healthcare plans, minimizing unexpected events that might contribute to mortality or additional burden of health complications highlights the importance of identification and assessment of variants in genes related to bleeding and coagulation. Conventional patient/symptom/variant approach has provided the basis for prevention, screening and diagnostic programs, and extending this knowledge to widen the scope of the investigated genes and variants can be very useful in management of anticipated health events.

Programs such as the SHGP are ideal for a population based application of genomic medicine. Patients benefit from clinical insights into their condition. Additionally, accumulated genomic data contributes a tremendous and valuable amount of knowledge to the entire community. Current medical records contain important and detailed clinical information that include thorough phenotyping, progress of condition and response to medication, so combining it with detailed genomic data will empower the health care provider with information that will ultimately lead to better healthcare.

In this study we used a set of 1285 individuals (P-cohort) diagnosed or recruited for molecular assessment of excessive bleeding or blood disorders using targeted NGS. We performed a focused variant analysis in 17 selected genes associated with blood or bleeding disorders. Then we looked at the frequency of these variants in our replication cohort (R-cohort) of randomly ethnically selected normal exomes.

Our primary filtering process retained a total of 821 variants, the majority of variants were present in *VWF*, followed by *F8*, *G6PD*, *F5*, and *F7*, while *FGG*, *FGB*, and *HBA1* had the lowest number of variants. The later genes have smaller transcripts and lower number of exomes compared to the genes with higher variant burden. These findings suggest that those genes with high number of variants may represent a pool that could be frequently mutated in our population, and can be the main target for molecular diagnosis in cases with no clear underlying genetic defect. However, if we focus on HGMD reported variants; *F8*, *VWF*, *G6PD*, and *F9* presented with higher count of HGMD variants, but when we calculated the percentage of HGMD variants/gene the following genes; *F9*, *HBB*, *HBA2*, and *F8* were flagged with a higher percentage of previously reported variants suggesting that these genes can be prioritized for direct diagnosis and carrier testing for both X-linked recessive and autosomal recessive conditions.

*VWF* reported the highest number of novel variants followed by *F5*, *F8*, and *G6PD*, however, when calculating the percentage of novel variants/ gene; *HBA2*, *F2*, *FGG*, *F13B*, and *FGA* had the highest percentage of novel variants. This renders them as the main genes for novel mutation discovery studies in our population.

There is an emphasis on the role of heterozygous carriers in AR disease that was the basis for the concept of premarital screening programs especially in consanguineous populations^26,27^. The concept is also included in prenatal diagnosis (PND) and preimplantation genetic diagnosis (PGD) for monogenic inherited disorders^28,29^, and expanded carrier testing (ECT), a term that can be extended to include screening for genes involved in complex or multigene disorders^30,31^.

Our analysis for heterozygous variants in AR genes identified 22 HGMD and 18 novel (pathogenic, likely pathogenic) heterozygous variants in undiagnosed and un affected individuals, which points out to these individuals as carries or cases with attenuated form of bleeding or clotting. *VWF*, *F10*, *F5*, and *F7* had the highest number of heterozygous variants. *VWF* had the highest number of HGMD heterozygous and novel variants, which is expected since Von Willebrand disease is the most common bleeding condition worldwide. These findings indicate that there is a mutational pressure for new and potential pathogenic variants in these genes. These genes should be prioritized for carrier testing and patient evaluation. Although β-Thalassemia is a common blood disorder, *HBB* gene had a lower number of variants than expected, this can be explained by the design of the panel, which doesn’t cover deep intronic, UTR and copy number variants, these are common types of causative mutations in β-Thalassemia^32–34^.

From our selected gene list only three genes are located on the X-chromosome and follow an X-linked inheritance; *F8*, *F9*, and *G6PD*. Both *F8* and *G6PD* accumulated a larger number and percentage of HGMD and novels variants, some of these variants were identified in males in a hemizygous state, which may require clinical reassessment of the case if the variant is classified pathogenic or likely pathogenic.

In summary, when we rank the genes based on the following three parameters; number of HGMD, novel variants, and total pathogenic mutations, *VWF*, *F8*, *and G6PD* emerge as the main genes of interest followed by *F7* and *F9*. If we use this data for designing of a national screening program or a patient oriented personalized assessment, the priority list of genes should include the following AR genes for carrier testing and identification of attenuated heterozygous cases with mild bleeding symptoms; *HBB*, *F7*, *F13A1*, and *F11* in addition to *VWF* which carried the highest mutational burden. Variants in these genes can be considered as contributors in unexplained bleeding disorders or as part of a management plan for patients who undergo surgery, dialysis, medical procedures, or treatments with potential hemorrhagic side effects.

In this exercise, we are trying to generate information that might be helpful for nationwide screening and prevention programs. The scientific literature is biased towards Caucasian populations and data from other ethnic groups is important in healthcare decisions, management, and drug development. In the past three decades public healthcare systems have benefited from the molecular data, and translated the knowledge in designing screening and national preventative programs some of which were highly successful in decreasing transmission^35,36^. Currently there are collaborative genome programs and population specific genetic initiatives around the world contributing and providing data to help decision makers, different health related government and commercial entities to direct diagnosis and treatment to adopt a precision medicine approach. This will not be achieved without acknowledging population differences, and generating data from different ethnic groups to make sure that health care and medication is not skewed towards the benefit of specific advantaged populations.

## Methods

### Cohorts and NGS

Two cohorts used in this study; the primary (investigation anonymous cohort, P-cohort) of 1285 cases and family members suspected of a hematological disease, mainly bleeding disorder. These cases were analyzed using targeted gene sequencing. The secondary (replication cohort, R-cohort) of a maximum of 5000 ethnically matched exomes from cases not related or diagnosed with bleeding or blood disorders and were sequenced as part of SHGP^17,37,38^. The frequency of the variants identified in the P-cohort was also determined in the secondary R-cohort.

All samples at the SHGP are consented with ethical approval that covers use of anonymized data for analysis. The specified samples used for this study (P-cohort) are part of an approved project (RAC#2130036) at King Faisal Specialist Hospital and Research Center (KFSH&RC), and in compliance with the 1964 Helsinki declaration and all its amendments.

The samples were sequenced using the Ion Torrent technologies (Thermo Fisher Scientific, Waltham, MA, USA). WES was based on the Ion Ampliseq protocol. The heme gene panel is a custom gene panel that was designed to capture 393 genes related to the hematology disorders. The sequencing protocol was described previously^17^, briefly, at least 10 ng of DNA was amplified using two primer pools that cover the coding sequence of the 393 SHGP heme panel genes using AmpliSeqHiFi mix. After PCR, the two products were pooled for each sample and digested using FuPa reagent, then ligated to the designated adapters and purified. This was followed by quantifications of the resulting liberties using qPCR. Samples concentrations were measured, normalized to 100 pM and pooled; 33samples/emulsion PCR using the Ion OneTouch system. The products were enriched using Ion OneTouch ES according to the manufacturer’s instructions. The resulting enriched template positive ion sphere particles were sequenced on the Ion Proton instrument (Thermo Fisher Scientific, Waltham, MA, USA).

### Selection of genes

The SHGP heme panel contains 393 genes that cover different nonmalignant common and rare blood and bleeding disorders. These disorders include anemias (aplastic and hemolytic), and excessive bleeding and clotting disorders. We selected 17 genes (Supplementary Table 8), 13 of which are linked to different bleeding disorders and four related to common blood disorders. We have selected the genes based on their prevalence globally and in the Saudi population^39–42^. Genes associated with Glanzmann Thrombasthenia, a rare genetic bleeding disorder, were excluded since they were evaluated in an earlier study^20^, whereby 72 individuals were screened and 17 mutations were identified in *ITGA2B* and *ITGB3*.

### Bioinformatics analysis

#### Analysis pipeline and variant selection

The analytical pipeline starts with base calling using the TorrentSuite package (Thermo Fisher Scientific, Waltham, MA, USA). For gene panels, the samples were de-multiplexed into multiple sequence files, each named after the sample barcode. The next step in the pipeline includes the alignment of the sample reads to the reference human genome (hg19), using the tmap program which is part of the TorrentSuite. Then the program TSVC, which is also part of the TorrentSuite package, is invoked to call the variants. The default parameters for germline variant calling was used to do the alignment and call the variants.

The variant call file (vcf), resulting from the variant calling step, was annotated using public, in-house, and commercial databases. We used the ANNOVAR package to annotate the variants with public databases for genes (RefSeq and Ensembl), population frequencies (ExAC, GnomAD), variant pathogenicity (PolyPhen, Sift, and Combined Annotation Dependent Depletion; CADD). On top of the ANNOVAR tracks, we also added OMIM and up-to-date version of ClinVar. We also used the commercial HGMD package. In addition, we annotated the variants with population frequencies from SHGP.

After annotation, the variants were filtered out to exclude synonymous and intronic variants and indels. Variants which are frequent in the population (>1%) and predicted by softwares to be benign were also filtered out. The remaining set of variants were then revised manually. The final set of the variants were then summarized and their frequencies within the investigation cohorts is computed and reported.

#### Variants selection and classification

To assess the pathogenicity of the variants, we grouped the variants into three groups: (1) Variants of known pathogenicity, (2) Novel variants, and (3) polymorphisms.

The first two groups were filtered as follows:

1. The variants of known pathogenicity are those that have been previously reported in the medical databases. We identified variants that are previously reported in HGMD and we used the ClinVar interpretations where possible (Accession date 27th April 2021). These variants were retained provided that they are related to the phenotype.
2. The novel variants are those that are not available in ClinVar and HGMD and available only in our P-cohort. To confirm the significance of these variants, we followed the ACMG guidelines: First, we confirmed that their frequency is less than 1% in the Saudi and international population frequency databases. Second, for non-synonymous variants we ensured that they may alter the protein structure by checking their CADD, PolyPhen, and Sift score (Sift < 0.05, PolyPhen > 0.95, and CADD > 20). Variants not matching these criteria were filtered out.

Two interrogative softwares that apply the ACMG criteria were used to confirm the classification of variants in these two groups^43–45^. As for polymorphisms; these are variants that are predicted or listed in dbSNP to be benign.

For the AR genes CF was calculated for a specific variant or gene as follows; number of heterozygotes individuals in the studied population divided by the total number of individuals. This was calculated for Pathogenic, likely pathogenic and VUS only.

### Reporting summary

Further information on research design is available in the Nature Research Reporting Summary linked to this article.

## Supplementary information

Supplementary Information

Reporting Summary

## Data Availability

Raw data was provided by Saudi Human Genome Program (SHGP) for research purposes at the following link (https://shgp.kacst.edu.sa/index.en.html). Institutional ethical approval and an application that specifies the scientific question for data mining are required. For any further information on the SHGP data availability policy please email SHGP; support@saudigenomeproject.com. The novel mutations have been deposited in Clinvar NCBI site (Accession numbersSCV001622445—SCV001622583). Furthermore, the variant files of this study that can be neither deposited to Clinvar nor formatted as supplementary tables have been made available via figshare (10.6084/m9.figshare.14785911) as per journal policy. Additional data will be available upon request for research purposes.

## Code availability

NGS data was analyzed using the following publicly available programs: 1. Alignment/Variant Calling: Ion Torrent—Torrent Suite (TS version 5.12) https://github.com/iontorrent/TS. 2. Annotation: Annovar System (https://annovar.openbioinformatics.org/en/latest/) [v 20210123].
