# Supplementary material for: Molecular classification of blood and bleeding disorder genes
Source: NPJ Genom Med. 2021 Jul 16;6:62. doi: 10.1038/s41525-021-00228-2 (PMC8285395; doi:10.1038/s41525-021-00228-2)
Supplement: Supplementary file 1 — Supplementary Information [file 41525_2021_228_MOESM1_ESM.pdf]

Supplementary File:

Molecular Classification of Blood and Bleeding Disorder Genes

Batoul Baz [1,2*], Mohamed Abouelhoda [1,2,3*], Tarek Owaidah[4], Majed Dasouki[2], Dorota Monies[1,2*], Nada AlTassan[1,2⊥]

Supplementary Table 1 Average QC values of the targeted sequenced samples

**Avg. number of reads**	2942878	**Average depth**	254
**Mean read length at Q0**	178	**Target coverage at 1X**	99.10%
**Mean read length at Q20**	145	**Target coverage at 10X**	98.21%
**Mean read length at Q30**	131	**Target coverage at 20X**	96.20%
**Percent reads on target**	97%	**Avg. no of variants/ sample**	1195
**Number of amplicons**	7472	**Ti/Tv**	2.35

Supplementary Table 2 Count and percentage of HGMD, Novel and polymorphisms in our target genes

	Count of variants in the selected genes																
	F2	***F5***	***F7***	***F8***	***F9***	***F10***	***F11***	***F13A***	***F13B***	***FGA***	***FGB***	***FGG***	***G6PD***	***HBA1***	***HBA2***	***HBB***	***VWF***
**HGMD**	1	2	8	23	13	1	4	2	0	1	0	1	13	0	5	8	16
**dbSNP**	18	50	19	37	11	19	17	25	15	15	12	4	22	0	3	8	78
**Novel**	29	32	23	47	5	16	11	14	18	18	2	7	31	4	13	7	93
**Total**	48	84	50	107	29	36	32	41	33	34	14	12	66	4	21	23	187
	**Percentage of variants**																
	F2	***F5***	***F7***	***F8***	***F9***	***F10***	***F11***	***F13A***	***F13B***	***FGA***	***FGB***	***FGG***	***G6PD***	***HBA1***	***HBA2***	***HBB***	***VWF***
**HGMD**	0.021	0.024	0.16	0.215	0.45	0	0.13	0.049	0	0.03	0	0.1	0.197	0	0.238	0.35	0.09
**dbSNP**	0.375	0.595	0.38	0.346	0.38	0.5	0.53	0.61	0.45	0.44	0.9	0.3	0.333	0	0.143	0.35	0.42
**Novel**	0.604	0.381	0.46	0.439	0.17	0.4	0.34	0.341	0.55	0.53	0.1	0.6	0.47	1	0.619	0.3	0.5
**Total**	1	1	1	1	1	1	1	1	1	1	1	1	1	1	1	1	1
**Breakdown of type of variant per gene**																	
	F2	***F5***	***F7***	***F8***	***F9***	***F10***	***F11***	***F13A***	***F13B***	***FGA***	***FGB***	***FGG***	***G6PD***	***HBA1***	***HBA2***	***HBB***	***VWF***
**LoF**	0	3	2	9	3	3	0	0	1	1	0	0	0	0	2	1	12
**Non Syn.**	13	37	20	37	11	14	9	13	7	21	3	6	19	0	7	5	60
**Intronic/UTR**	26	25	20	43	11	12	18	18	18	9	6	6	39	4	9	15	75
**Syn**	9	19	8	18	4	7	5	10	7	3	5	0	8	0	3	2	40
**Total**	**48**	**84**	**50**	**107**	**29**	**36**	**32**	**41**	**33**	**34**	**14**	**12**	**66**	**4**	**21**	**23**	**187**

LoF; loss of function, NonSyn: Non synonymous, Syn: Synonymous.

Supplementary Table 3 HGMD reported variants identified in our cohorts

Gene	Type	AA change	HGMD2020	P-Cohort	R-cohort	dbSNP (build 154)	CADD	ClinVar	ACMG Classification	P-CF	R-CF
F5	nonsyn.	NM_000130:exon25:c.6604C>T:p.Arg2202Cys	CM105385/ Factor V deficiency	0.001556*	0	rs754980174	35	NP	VUS	0.00000	0.00000
F5	nonsyn.	NM_000130:exon7:c.1001G>C:p.Arg334Thr	CM980658/ Thrombosis	0.002335⊥	0.000437⊥	rs118203906	22.9	Pathogenic	Likely Benign	0.00233	0.000437
FGA	nonsyn.	NM_000508:exon5:c.1880G>T:p.Arg627Leu	CM180606/ Hypofibrinogenaemia	0.000778⊥	0	rs762123879	11.82	NP	Likely Benign	0.00078	0.00000
FGG	nonsyn.	NM_000509:exon7:c.709T>C: p.YTyr237His	CM144380/ Hypofibrinogenaemia	0.000778⊥	0	rs762488338	28	NP	Likely Pathogenic	0.00078	0.00000
F11	splice	NM_000128:exon4:c.325+5G>T	CS131881/ Factor XI deficiency	0.000778⊥	0	rs372102736		VUS	VUS	0.00078	0.00000
F11	nonsyn.	NM_000128:exon8:c.797G>A: p.S266Asn	CM980652/ Factor XI deficiency	0.001556⊥	0.000936⊥	rs145168351	18.45	NP	Likely Benign	0.00156	0.00090
F11	nonsyn.	NM_000128:exon8:c.803G>A: p.Arg268His	CM083501/ Factor XI deficiency	0.000778⊥	0	rs201688862	23.3	VUS	Likely Benign	0.00078	0.00000
F11	nonsyn.	NM_000128:exon15:c.1718G>A:p.Gly573Glu	CM043486/ Factor XI deficiency	0.000778⊥	0	rs754109115	27.1	VUS	VUS	0.00078	0.00000
F13A1	nonsyn.	NM_000129:exon12:c.1622G>A:p.Arg541Gln	CM103181/ Factor XIII deficiency	0.001556⊥	0	rs367679357	16.59	NP	VUS	0.00156	0.00000
F13A1	nonsyn.	NM_000129:exon3:c.233G>A: p.Arg78His	CM040029/ Factor XIII deficiency	0.000778*	0.000312⊥	rs768024997	33	NP	Likely Pathogenic	0.00000	0.00030
F13A1	splice	NM_000129:exon6:c.798+2T>C	NP	0.000778^	0.000312⊥		25.1	Pathogenic/ RCV001092974	Pathogenic	0.00078	0.0003
F2	nonsyn.	NM_000506:exon9:c.1117G>A:p.Gly373Ser	CM002956/ Prothrombin deficiency	0.000778⊥	0.000218⊥	rs773672109	24	NP	Likely Pathogenic	0.00078	0.0002
HBB	nonsyn.	NM_000518:exon3:c.404T>C: p.Val135Aal	CM015851/ Haemoglobin variant	0.000778⊥	0	rs33966761	23.3	Pathogenic/ RCV000016521.4	Likely Pathogenic	0.00078	0.00000
HBB	nonsyn.	NM_000518:exon3:c.380T>A: p.Val127Glu	CM850034/	0.000778⊥	0	rs33925391	19.57	Pathogenic/likely	VUS	0.00078	0.00000

			Haemoglobin variant					Pathogenic/RCV001260263			
HBB	splice	NM_000518:exon2:c.315+1G>A	CS820006/Beta thalassaemia	0.004669*⊥	0.002183⊥	rs33945777	24.8	Pathogenic/ RCV001170018	Pathogenic	0.00389	0.00040
HBB	splice	NM_000518:exon1:c.92+6T>C	CS820004/Beta thalassaemia	0.001556⊥	0.000312⊥	rs35724775		Pathogenic/ RCV000507521	Likely Pathogenic	0.00156	0.00000
HBB	nonsyn.	NM_000518:exon1:c.92G>C:p.Arg31Thr	CS890123/Beta thalassaemia	0.002335⊥	0	rs33960103	31	Pathogenic/ RCV000507793	Pathogenic	0.00000	0.00000
HBB	fs.Ins	NM_000518:exon1:c.27dupG: p.Ser10fs	CI931090/ Beta thalassaemia	0.006226*⊥	0	rs35699606		Pathogenic/ RCV001001672	Pathogenic	0.00156	0.00000
HBB	splice	NM_000518:c.-31C>T	CR0911017/ Beta thalassaemia	0.000778⊥	0	rs63750628		Benign/VUS/ RCV000029956	VUS	0.00078	0.00000
HBB	UTR	chr11:g.5248343:G>C	CR106856/Beta thalassaemia|	0.002335⊥	0	rs397515291		VUS/ RCV000780306	VUS	0.00000	0.00000
VWF	nonsyn.	NM_000552:exon52:c.8327C>T:p.Pro2776Leu	CM163370/ Von Willebrand disease 1	0.000778⊥	0	rs61751312	25.7	NP/ RCV000086919	Likely Pathogenic	0.00078	0.00000
VWF	nonsyn.	NM_000552:exon45:c.7604G>A:p.Arg2535Gln	CM143307/ Von Willebrand disease 3	0.001556⊥	0	rs137987906	26.7	NP	Likely Benign	0.00156	0.00000
VWF	nonsyn.	NM_000552:exon44:c.7493C>A:p.Ala2498Asp	CM1714149/ Von Willebrand disease	0.003113⊥	0.000624⊥	rs369669154	25.8	Likely Pathogenic /RCV000851866	Likely Pathogenic	0.00311	0.00000

VWF	nonsyn.	NM_000552:exon43:c.7390C>T:p.Arf2464Cys	CM070317/ Von Willebrand disease 1	0.000778⊥	0	rs61751286	34	Pathogenic/ Likely Pathogenic/ RCV001270490	Likely Pathogenic	0.00078	0.00000
VWF	nonsyn.	NM_000552:exon37:c.6433C>T:p.Pro2145Ser	CM070306/Von Willebrand disease 1	0.017121⊥	0.01466⊥	rs61750618	0.006	VUS/ RCV000778376	Likely Benign	0.01712	0.00500
VWF	nonsyn.	NM_000552:exon28:c.4789C>T:p.Arg1597Trp	CM890116/Von Willebrand disease 2a	0.002335⊥	0	rs61750117	25.8	Pathogenic/ RCV000086797	Pathogenic	0.00233	0.00000
VWF	stopgain	NM_000552:exon28:c.4696C>T:p.Arg1566Ter	CM061230/Von Willebrand disease	0.000778*	0	rs61750112	37	Pathogenic/RCV000086788	Pathogenic	0.00000	0.00000
VWF	nonsyn.	NM_000552:exon28:c.4414G>C:	HM070121/ Altered ristocetin-induced platelet aggregation	0.187549*⊥	0.129133*⊥	rs1800383	0.003	Likely benign/ RCV000397126.1	Likely benign	0.00000	0.07560
VWF	nonsyn.	NM_000552:exon28:c.4414G>C: p.Asp1472His	HM070121/ Altered ristocetin-induced platelet aggregation	0.002335*	0.129133*⊥	rs1800383	0.003	Likely_benign/ RCV000397126.1	Likely benign	0.00000	0.07560
VWF	nonsyn.	NM_000552:exon28:c.4195C>T:p.Arg1399Cys	CM095099/Von Willebrand disease 2m	0.001556⊥	0	rs61750077	31	Likely pathogenic/ RCV000852119	VUS	0.00156	0.00000
VWF	nonsyn.	NM_000552:exon28:c.3692A>G:p.Asn1231Ser	CM1714114/ Von Willebrand disease 1h	0.001556⊥	0.001248⊥	rs61749368	7.729	Likely pathogenic/ RCV001254138	Benign	0.00156	0.0012
VWF	splice	NM_000552:exon25:c.3379+1G>A	CS992483/ Von Willebrand, Normandy variant	0.000778*	0	rs2363337	23.7	Pathogenic/ RCV000851637	Pathogenic	0.00000	0.00000

VWF	nonsyn.	NM_000552:exon25:c.3281T>C:p.Ile1094Thr	CM070313/Von Willebrand disease 1	0.001556⊥	0.000936⊥	rs267607317	23.9	NP/ RCV000086644	Likely Benign	0.00156	0.0009
VWF	nonframe shift deletion	NM_000552:exon23:c.3101_3103del:p.1034_1035del	CD061485/Von Willebrand disease 2m	0.001556*⊥	0.00156*⊥	rs368366214		VUS/ RCV000988775	Benign	0.00078	0.00060
VWF	stopgain	NM_000552:exon7:c.813C>A:p.Tyr271Ter	BM1151814/Von Willebrand disease 1	0.003891*⊥	0		35	NP	Likely Benign	0.00233	0.00000
VWF	stopgain	NM_000552:exon3:c.100C>T:p.Arg34Ter	CM061228/Von Willebrand disease 3	0.000778*	0	rs61753984	35	NP/ RCV000086554	Pathogenic	0.00000	0.00000
F7	Intronic	NM_001267554:c.-30A>C	CR002894/ Factor VII deficiency	0.000778⊥	0	rs539578931		Likely pathogenic RCV000851580/	VUS	0.00078	0.00000
F7	nonsyn.	NM_001267554:exon3:c.214G>A:p.Gly72Ser	CM010247/ Factor VII deficiency|	0.000778^	0	rs563972504	25.9	NP	Likely Pathogenic	0.00078	0.00000
F7	nonsyn.	NM_001267554:exon3:c.260C>T:p.Ser87Phe	CM090323/ Factor VII deficiency	0.000778⊥	0.000936⊥	rs143855920	8.784	NP	Likely Pathogenic	0.00078	0.00009
F7	nonsyn.	NM_001267554:exon4:c.347T>G:p.Ile116Ser	CM034413/ Factor VII deficiency|	0.000778⊥	0.000312⊥	rs141219108	16.98	NP	Likely Pathogenic	0.00078	0.00031
F7	nonsyn.	NM_001267554:exon5:c.544G>A:p.Ala182Thr	CM001149/ Factor VII deficiency	0.000778⊥	0	rs764807079	17.61	NP	Likely Pathogenic	0.00078	0.00000
F7	nonsyn.	NM_001267554:exon6:c.658G>A:p.Ala220Thr	CM002766/ Factor VII deficiency	0.000778⊥	0	rs773627551	25	VUS/RCV001111142	Likely Pathogenic	0.00078	0.00000
F7	nonsyn.	NM_001267554:exon6:c.659C>T:p.Ala220Val	CM960528/ Disease/Factor VII deficiency	0.000778⊥	0.000936⊥	rs121964931	24.7	Likely Pathogenic/ Pathogenic/ RCV000852243	Pathogenic	0.00078	0.00093
F7	nonsyn.	NM_001267554:exon6:c.838C>T:p.Arg280Trp	CM940394/ Factor VII deficiency	0.000778⊥	0	rs750980786	26.8	NP	VUS	0.00078	0.00000
F10	nonsyn.	NM_001312674:exon7:c.904C>T:p.Arg302Cys	CM980649/ Factor X deficiency	0.000778⊥	0	rs755110383	24.4	NP	VUS	0.00078	0.00000

HBA2	nonsyn.	NM_000517:exon3:c.409C>A:p.Leu137Met	CM940913/ Haemoglobin variant	0.000778⊥	0	rs41364652	24.6	VUS/ RCV000508426	Likely Pathogenic	NA	NA
HBA2	stoploss	NM_000517:exon3:c.427T>C:p.ter143Gln	CM880037/Haemoglobin variant	0.001556⊥	0	rs41464951	22.3	Pathogenic/ RCV000985726	Pathogenic	NA	NA
HBA2	splice	NM_000517:c.*92A>G	CR920785/ Alpha Thalassaemia	0.002335⊥	0.001766⊥	rs63750067		Pathogenic/RCV000507591	Likely Pathogenic	NA	NA
HBA2	splice	NM_000517:c.*94A>G	CR830007/Alpha Thalassaemia	0.004669*⊥	0.002943*⊥	rs63751269		Pathogenic /RCV000417217.2	VUS	NA	NA
HBA2	UTR	chr16:g.223735:A>G	CR042844/ Haemoglobin variant	0.834241*⊥	0.639788*⊥	rs2685121		Benign/ RCV001078238	Benign	NA	NA
F9	nonsyn.	NM_000133:exon1:c.8G>A:p.Arg3His	CM940509/ Haemophilia B	0.001556^	0	rs148060786	5.973	Benign/ RCV001081775	Benign	NA	NA
F9	frameshift deletion	NM_000133:exon2:c.252delA:p.Thr85LeufsTer19	CD910506/ Haemophilia B	0.001556*	0			NP	Likely Pathogenic	NA	NA
F9	Intronic	chrX:g.138623208:A>G	CS157328/ Haemophilia B	0.005447*⊥^	0.004414*⊥^	rs3134809		NP	Benign	NA	NA
F9	nonsyn.	NM_001313913:exon5:c.466A>G:p.Thr156Ala	CM157326/Deep vein thrombosis, reduced risk	0.247471*⊥	0.198058*⊥	rs6048	0.002	Benign/ RCV000396693	Benign	NA	NA
F9	nonsyn.	NM_001313913:exon7:c.741G>C:p.Glu247Asp	CM173020/ Haemophilia B	0.000778*	0		1.2	VUS/ RCV001028011	VUS	NA	NA
F9	nonsyn.	NM_001313913:exon7:c.848T>C:p.Leu283Pro	CM940611/ Haemophilia B	0.000778*	0		26.9	NP	Likely Pathogenic	NA	NA
F9	nonsyn.	NM_001313913:exon7:c.853G>A:p.Glu285Lys	CM045765/ Haemophilia B	0.000778⊥	0.000883*⊥	rs150351950	0.217	Benign / RCV000861890	Benign	NA	NA

F9	nonsyn.	NM_001313913:exon7:c.911C>T:p.Thr304Met	CM940625/ Haemophilia B	0.001556*^	0	rs137852254	32	Pathogenic/RCV000792734	Pathogenic	NA	NA
F9	nonsyn.	NM_001313913:exon7:c.1022G>A:p.Arg341Gln	CM940660/ Haemophilia B	0.005447*⊥	0	rs137852259	25.5	Pathogenic/ RCV000851995	Pathogenic	NA	NA
F9	stopgain	NM_001313913:exon7:c.1179G>A:p.Trp393Ter	CM940718/ Haemophilia B	0.001556*	0		41	NP	Pathogenic	NA	NA
F9	nonsyn.	NM_001313913:exon7:c.1232G>A:p.Arg411Gln	CM940739/ Haemophilia B	0.000778*	0.000294⊥*	rs143018900	15.82	NP	VUS	NA	NA
F9	stopgain	NM_001313913:exon7:c.1244G>A:p.Trp415Ter	CM940744/ Haemophilia B	0.000778*	0		40	NP	Pathogenic	NA	NA
G6PD	nonsyn.	NM_000402:exon12:c.1478G>A:p.Arg493His	CM910164/Glucose-6-phosphate dehydrogenase deficiency	0.000778⊥^	0.000531*⊥^	rs72554664	32	Pathogenic/ RCV000823393	Likely Pathogenic	NA	NA
G6PD	splice	NM_000402:exon12:c.1455-13T>C	CS1811383/Glucose-6-phosphate dehydrogenase deficiency	0.505058*⊥	0.366463*⊥	rs2071429		Benign/Likely_benign	Benign	NA	NA
G6PD	nonsyn	NM_000402:exon11:c.1401C>T:p.Tyr467Tyr	CM133486/ Glucose-6-phosphate dehydrogenase deficiency	0.32607*⊥	0.249801*⊥	rs2230037		Likely Benign/RCV000011084	Benign	NA	NA
G6PD	nonsyn.	NM_000402:exon9:c.1093G>A:p.Ala365Thr	CM880033/ Glucose-6-phosphate dehydrogenase deficiency	0.000778*^	0.001594*⊥^	rs5030869	27.3	Pathogenic/ RCV000180546.4	Likely Pathogenic	NA	NA
G6PD	nonsyn.	NM_000402:exon9:c.961G>A:p.Val321Met	CM930275/Glucose-6-phosphate dehydrogenase deficiency	0.000778⊥^	0	rs137852327	27.7	Pathogenic RCV000405688/	Pathogenic	NA	NA
G6PD	nonsyn.	NM_000402:exon6:c.724A>G:p.Met242Val	CM930271/ Glucose-6-phosphate dehydrogenase deficiency	0.000778⊥	0.000531⊥	rs782754619	23.3	NP	Likely Pathogenic	NA	NA

G6PD	nonsyn.	NM_000402:exon6:c.653C>T: p.Ser218Phe	CM880032/Glucose-6-phosphate dehydrogenase	0.031128*⊥^	0.026043*⊥^	rs5030868	24.2	Pathogenic/ RCV000445579	Likely Pathogenic	NA	NA
G6PD	nonsyn.	NM_000402:exon6:c.632A>T: p.Asp211Val	CM930270/Glucose-6-phosphate dehydrogenase deficiency	0.000778⊥	0	rs5030872	12.06	NP	Pathogenic	NA	NA
G6PD	nonsyn.	NM_000402:exon6:c.577G>A: p.Gly193Ser	CM890050/ Glucose-6-phosphate dehydrogenase deficiency	0.000778⊥	0	rs137852314	33	Pathogenic/ RCV000991016	Pathogenic	NA	NA
G6PD	nonsyn.	NM_000402:exon5:c.567G>C: p.Met189Ile	CM103353/ Glucose-6-phosphate dehydrogenase deficiency	0.000778⊥	0.000266⊥	rs370918918	18.24	VUS/ RCV001285295	VUS	NA	NA
G6PD	nonsyn.	NM_000402:exon5:c.466A>G: p.Gln156Asp	CM880030/ Glucose-6-phosphate dehydrogenase deficiency	0.035019*⊥	0.025777*⊥	rs1050829	0.037	NP	Likely Benign	NA	NA
G6PD	nonsyn.	NM_000402:exon4:c.292G>A: p.Val98Met	CM880029/Glucose-6-phosphate dehydrogenase deficiency	0.010117*⊥^	0.007175*⊥^	rs1050828	27.2	NP	VUS	NA	NA
G6PD	nonsyn.	NM_000402:exon3:c.233T>C: p.Ile78Thr	CM930263/ Glucose-6-phosphate dehydrogenase deficiency	0.026459*⊥^	0.018602*⊥^	rs76645461	18.09	Pathogenic/ RCV000224890	Likely Pathogenic	NA	NA
F8	nonsyn.	NM_019863:exon5:c.562C>T: p.Arg188Cys	CM910153/ Haemophilia A	0.000778*^	0	rs137852473	25.5	Likely Pathogenic/ RCV000757257.1	Likely Pathogenic	NA	NA
F8	nonsyn.	NM_019863:exon4:c.364A>G: p.Met122Val	CM950422/ Haemophilia A	0.029572*⊥	0.017363*⊥	rs1800297	7.091	Benign/RCV000033894.3	Benign	NA	NA
F8	stopgain	NM_019863:exon3:c.277C>T: p.Arg93Ter	CM850003/ Haemophilia A	0.000778*^	0	rs137852355	47	Pathogenic/ RCV000011038.3	Pathogenic	NA	NA

F8	nonsyn.	NM_019863:exon2:c.140G>A:p.Arg47His	CM940403/ Haemophilia A	0.000778*^	0	rs137852466	35	Pathogenic/RCV000011033.4	Pathogenic	NA	NA
F8	nonsyn.	NM_000132:exon19:c.6107A>G:p.Tyr2036Cys	CM011333/ Haemophilia A	0.003113*	0		26.2	NP	Likely Pathogenic	NA	NA
F8	nonsyn.	NM_000132:exon19:c.5999G>T:p.Gly2000Val	CM103956/ Haemophilia A	0.000778*	0		33	Pathogenic/ RCV001000840	Pathogenic	NA	NA
F8	nonsyn.	NM_000132:exon16:c.5542G>A:p.Glu1848Lys	CM083683/ Haemophilia A	0.000778*	0		33	NP	Likely Pathogenic	NA	NA
F8	nonsyn.	NM_000132:exon16:c.5411T>C:p.Phe1804Ser	CM031986/ Haemophilia A	0.000778*	0		29	NP	Likely Pathogenic	NA	NA
F8	nonsyn.	NM_000132:exon15:c.5252A>G:p.Lys1751Arg	CM082628/ Haemophilia A	0.000778*	0		27.2	NP	Likely Pathogenic	NA	NA
F8	nonsyn.	NM_000132:exon14:c.5218A>G:p.Arg1740Gly	CM020426/ Haemophilia A	0.000778*	0		13.58	NP	Pathogenic	NA	NA
F8	stopgain	NM_000132:exon14:c.5143C>T:p.Arg1715Ter	CM900094/ Haemophilia A	0.000778*^	0	rs137852439	43	Pathogenic/ RCV000010980	Pathogenic	NA	NA
F8	nonsyn.	NM_000132:exon14:c.4531G>A:p.Val1511Ile	CM082634/ Haemophilia A	0.001556*	0.000294*⊥	rs186353717	12.48	NP	Likely Benign	NA	NA
F8	stopgain	NM_000132:exon14:c.4363C>T:p.Gln1455Ter	CM076177/ Haemophilia A	0.000778*	0		35	NP	Pathogenic	NA	NA
F8	nonsyn.	NM_000132:exon14:c.3780C>G:p.Asp1260Glu	CM960556/ Decreased factor VIII activity	0.20856*⊥	0.177163*⊥	rs1800291	0.002	Benign	Likely Benign	NA	NA
F8	nonsyn.	NM_000132:exon14:c.3169G>A:p.Glu1057Lys	CM910136/ Haemophilia A	0.000778⊥	0	rs28933673	23.2	Likely Pathogenic/VUS/ RCV000010964	VUS	NA	NA
F8	nonsyn.	NM_000132:exon14:c.2994T>G:p.His998Gln	CM062651/ Haemophilia A	0.000778*	0.001471*⊥	rs149853218	0.001	Likely benign/ RCV000991024	Likely Benign	NA	NA
F8	nonsyn.	NM_000132:exon12:c.1835G>C:p.Arg612Pro	CM095522/ Haemophilia A	0.003113*	0		24.8	NP	Likely Pathogenic	NA	NA

F8	nonsyn.	NM_000132:exon10:c.1508G>A:p.Arg503His	CM014185/ Haemophilia A	0.001556⊥	0.001177*⊥	rs35383156	26	Likely_benign/ RCV000991025	Benign	NA	NA
F8		NM_000132:exon9:c.1443+18T>G	CS055579/ Haemophilia A	0.001556*⊥	0.000883*⊥	rs782583179		Benign / RCV001258295	Benign	NA	NA
F8	nonsyn.	NM_000132:exon6:c.760A>G: p.Asn254Asp	CM095521/ Haemophilia A	0.004669*	0		26	NP	Likely Pathogenic	NA	NA
F8	nonsyn.	NM_000132:exon4:c.396A>C: p.Glu132Asp	CM980667/ Haemophilia A	0.000778⊥	0	rs137852388	14.71	Pathogenic/RCV000010884	VUS	NA	NA
F8		NM_000132:exon3:c.266-19G>A	CS1314199/ Haemophilia A	0.017121*⊥	0.012066*⊥	rs28370201		Benign/ RCV000991028	Benign	NA	NA
F8	nonsyn.	NM_000132:exon2:c.230T>C: p.Leu77Pro	CM053251/ Haemophilia A	0.001556*	0.001471⊥		22.3	NP	VUS	NA	NA

⊥ heterozygous, *homozygous/hemizygous males (X-chromosome genes) , *⊥ heterozygous and homozygous/ hemizygous males (X-chromosome genes). ^ Recorded as hemizygous in at least one male individual.

Nonsyn: nonsynonymous. fs.Ins: Frameshift insertion. P-cohort: Primary cohort R-cohort: Replication cohort (value= Case number/Total Cases). NA: Non Available, NP: not provided. CADD: Combined Annotation Dependent Depletion.

Supplementary Table 4 Novel variants identified in our cohorts

Gene	Type	AA change	P-Cohort	R-Cohort	CADD	ACMG Classification	P-CF	R-CF
F5	nonsyn.	NM_000130:exon21:c.6010T>C:p.Trp2004Arg	0.000778*	0	29.2	VUS	0	0
F5	nonsyn.	NM_000130:exon17:c.5594T>G:p.Leu1865Arg	0.000778[⊥]	0.000218[⊥]	29.6	VUS	0.000778	0
F5	fs.del	NM_000130:exon15:c.5022delA:p.Gly1674fs	0.003113*[⊥]	0.00131*[⊥]	NA	VUS	0.001556	0.0004
F5	nonsyn.	NM_000130:exon13:c.4297C>T:p.Leu1433Phe	0.001556[⊥]	0.000437[⊥]	4.543	VUS	0.001556	0
F5	nonsyn.	NM_000130:exon13:c.3995T>C:p.Leu1332Pro	0.004669*[⊥]	0.001528*[⊥]	0.119	VUS	0.003891	0
F5	nonsyn.	NM_000130:exon13:c.3674C>A:p.Ala1225Asp	0.005447[⊥]	0.001965[⊥]	0.002	Likely Benign	0.005447	0.0004
F5	fs.del	NM_000130:exon13:c.3576delG:p.Val1193Ter	0.000778[⊥]	0.000218[⊥]	NA	Likely Pathogenic	0	0.0002
F5	nonsyn.	NM_000130:exon13:c.2837G>T:p.Arg946Ile	0.000778[⊥]	0.000218[⊥]	19.88	VUS	0.000778	0
F5	fs.del	NM_000130:exon13:c.2615delG:p.Arg872LysfsTer12	0.000778*	0	NA	Pathogenic	0	0
F5	nonsyn.	NM_000130:exon13:c.2234A>G:p.Asn745Ser	0.000778[⊥]	0	0.009	VUS	0.000778	0
F5	nonsyn.	NM_000130:exon13:c.2232G>C:p.Leu744Phe	0.000778[⊥]	0	15.49	VUS	0.000778	0
F5	nonsyn.	NM_000130:exon13:c.1990A>G:p.Thr664Ala	0.000778[⊥]	0.001092[⊥]	18.66	VUS	0.000778	0.0008
F5	nonsyn.	NM_000130:exon4:c.430C>A:p.Pro144Thr	0.000778[⊥]	0.000218[⊥]	24.2	VUS	0.000778	0
F13B	nonsyn.	NM_001994:exon10:c.1693G>A:p.Ala565Thr	0.000778[⊥]	0.000624[⊥]	14.74	VUS	0.000778	0.0002
F13B	nonsyn.	NM_001994:exon6:c.881A>T:p.His294Leu	0.000778[⊥]	0	24.4	VUS	0.000778	0
F13B	nonsyn.	NM_001994:exon5:c.779G>T:p.Trp260Leu	0.000778[⊥]	0	34	VUS	0.000778	0
F13B	nonsyn.	NM_001994:exon4:c.625A>G:p.Thr209Ala	0.001556	0.000312[⊥]	0.045	VUS	0.001556	0
F13B	stopgain	NM_001994:exon4:c.565G>T:p.Gly189Ter	0.000778[⊥]	0	36	Pathogenic	0.000778	0
F13B	nonsyn.	NM_001994:exon4:c.521A>T:p.Asp174Val	0.000778[⊥]	0	25.1	VUS	0.000778	0
F13B	nonsyn.	NM_001994:exon3:c.431C>T:p.Pro144Leu	0.000778[⊥]	0.000312[⊥]	29.4	VUS	0.000778	0
FGA	nonsyn.	NM_000508:exon6:c.2527T>C:p.Trp843Arg	0.000778[⊥]	0	25.8	VUS	0.000778	0
FGA	nonsyn.	NM_000508:exon6:c.2372A>T:p.Asp791Val	0.000778[⊥]	0	28	VUS	0.000778	0
FGA	fs.del	NM_000508:exon6:c.2155delC:p.Gln719fs	0.000778*	0.000195*[⊥]	NA	Pathogenic	0	0
FGA	nonsyn.	NM_000508:exon6:c.2128G>A:p.Gly710Ser	0.000778[⊥]	0	32	VUS	0.000778	0
FGA	nonsyn.	NM_000508:exon6:c.2024T>G:p.Ile675Ser	0.000778*	0.000195[⊥]	27.2	VUS	0	0.0002
FGA	nonsyn.	:NM_000508:exon6:c.2024T>C:p.Ile675Thr	0.000778*	0.000195[⊥]	26	VUS	0	0.0002
FGA	nonsyn.	NM_000508:exon6:c.2021T>C:p.Leu674Ser	0.000778[⊥]	0	26.5	VUS	0.000778	0
FGA	nonsyn.	NM_021871:exon5:c.1930C>G:p.Pro644Ala	0.000778[⊥]	0	0.044	VUS	0.000778	0
FGA	nonsyn.	NM_021871:exon5:c.1915A>G:p.Lys639Glu	0.000778[⊥]	0.000389[⊥]	4.873	VUS	0.000778	0.0003
FGA	nonsyn.	NM_000508:exon5:c.1470C>G:p.Asp490Glu	0.000778[⊥]	0.000779*[⊥]	20.4	VUS	0.000778	0.0004

FGA	nonsyn.	NM_000508:exon5:c.1463G>A:p.Gly488Asp	0.000778⊥	0.000779*⊥	24.8	VUS	0.000778	0.0004
FGA	nonsyn.	NM_000508:exon5:c.829T>C:p.Tyr277His	0.000778⊥	0.000195⊥	0.004	VUS	0.000778	0.0002
FGG	nonsyn.	NM_000509:exon9:c.1202G>A:p.Arg401Gln	0.000778⊥	0.000312⊥	30	VUS	0.000778	0.0003
FGG	nonsyn.	NM_000509:exon7:c.700T>C:p.Trp234Arg	0.000778⊥	0	27.8	VUS	0.000778	0
FGG	nonsyn.	NM_000509:exon7:c.694A>G:p.Lys232Glu	0.000778⊥	0	26.4	VUS	0.000778	0
FGG	nonsyn.	NM_000509:exon6:c.606G>C:p.Gln202His	0.000778⊥	0	24.2	VUS	0.000778	0
F11	nonsyn.	NM_000128:exon4:c.230T>C:p.Val77Ala	0.000778⊥	0.003119⊥	23.4	VUS	0.000778	0.0018
F13A1	nonsyn.	NM_000129:exon14:c.1909G>A:p.Val637Ile	0.000778⊥	0	22.8	VUS	0.000778	0
F13A1	nonsyn.	NM_000129:exon12:c.1531G>A:p.Gly511Ser	0.001556⊥	0	7.012	VUS	0.001556	0
F13A1	nonsyn.	NM_000129:exon11:c.1346G>C:p.Gly449Ala	0.000778⊥	0	23.6	VUS	0.000778	0
F13A1	nonsyn.	NM_000129:exon10:c.1297T>G:p.Phe433Val	0.000778*	0	25	VUS	0	0
F13A1	nonsyn.	NM_000129:exon6:c.787G>A:p.Gly263Arg	0.001556*	0	28.6	VUS	0	0
F13A1	nonsyn.	NM_000129:exon3:c.186G>C:p.Lys62Asn	0.000778*	0	24.3	VUS	0	0
F2	nonsyn.	NM_000506:exon7:c.607A>G:p.Ser203Gly	0.001556⊥	0	0.001	VUS	0.001556	0
F2	nonsyn.	NM_000506:exon8:c.954T>G:p.Ser318Arg	0.000778*	0.000218⊥	0.064	VUS	0	0.0002
F2	nonsyn.	NM_000506:exon8:c.995G>C:p.Gly332Ala	0.000778⊥	0	23.8	Likely Pathogenic	0.000778	0
F2	nonsyn.	NM_000506:exon9:c.1070A>G:p.Glu357Gly	0.000778⊥	0.000218⊥	13.77	Likely Pathogenic	0.000778	0.0002
F2	splice	NM_001311257:exon10:c.1083-5C>T	0.000778⊥	0	NA	VUS	0.000778	0
F2	nonsyn.	NM_000506:exon10:c.1270G>A:p.Val424Met	0.000778⊥	0	31	Likely Pathogenic	0.000778	0
F2	nonsyn.	NM_000506:exon12:c.1496G>A:p.Gly499Glu	0.000778⊥	0	28.9	Likely Pathogenic	0.000778	0
F2	nonsyn.	NM_000506:exon12:c.1598G>A:p.Arg533Gln	0.000778⊥	0.000218⊥	14.45	VUS	0.000778	0.0002
HBB	nonsyn.	NM_000518:exon3:c.384G>C:p.Gln128His	0.000778⊥	0.000312⊥	15.02	VUS	0.000778	0.0003
VWF	nonsyn.	NM_000552:exon52:c.8401G>A:p.Ala2801Thr	0.001556⊥	0.000936⊥	26.7	VUS	0.001556	0.0009
VWF	nonsyn.	NM_000552:exon51:c.8195A>G:p.Gln2732Arg	0.000778⊥	0	15.55	VUS	0.000778	0
VWF	nonsyn.	NM_000552:exon45:c.7712G>A:p.Cys2571Tyr	0.000778*	0	26.2	VUS	0	0
VWF	nonsyn.	NM_000552:exon42:c.7201T>G:p.Ser2401Ala	0.085603*⊥	0.031192*⊥	20.2	VUS	0.000778	0.00031
VWF	nonsyn.	NM_000552:exon42:c.7114C>T:p.Pro2372Ser	0.000778⊥	0	19.34	VUS	0.000778	0
VWF	nonsyn.	NM_000552:exon38:c.6677C>T:p.Ser2226Phe	0.001556*⊥	0.001248*⊥	21.4	VUS	0.000778	0.0002
VWF	nonsyn.	NM_000552:exon37:c.6455A>G:p.Lys2152Arg	0.000778*	0.000312*⊥	22.5	VUS	0	0.0003
VWF	nonsyn.	NM_000552:exon37:c.6449G>C:p.Cys2150Ser	0.000778*	0.000312*⊥	27.8	VUS	0	0.0003
VWF	nonsyn.	NM_000552:exon37:c.6446A>G:p.Glu2149Gly	0.000778*	0.000312*⊥	24.8	VUS	0	0.0003
VWF	nonsyn.	NM_000552:exon36:c.6136A>G:p.Ile2046Val	0.000778⊥	0	6.731	VUS	0.000778	0
VWF	nonsyn.	NM_000552:exon36:c.6123C>A:p.Asp2041Lys	0.000778⊥	0.000312⊥	0.047	VUS	0.000778	0.0003
VWF	nonsyn.	NM_000552:exon34:c.5765G>A:p.Gly1922Glu	0.000778⊥	0.000312⊥	22.8	VUS	0.000778	0.0003

VWF	nonsyn.	NM_000552:exon34:c.5729C>T:p.Thr1910Ile	0.000778⊥	0	24.2	VUS	0.000778	0.0003
VWF	nonsyn.	NM_000552:exon34:c.5694G>C:p.Gln1898His	0.000778⊥	0.000312⊥	24.9	VUS	0.000778	0.0003
VWF	nonsyn.	NM_000552:exon28:c.5029A>G:p.Ile1677Val	0.002335⊥	0.000936⊥	0.001	VUS	0.002335	0.0009
VWF	nonsyn.	NM_000552:exon28:c.4922G>A:p.Arg1641lys	0.000778⊥	0	0.004	VUS	0.000778	0
VWF	fs.del	NM_000552:exon28:c.4413delC:p.Asp1472ThrfsTer53	0.005447*	0.004991⊥	NA	Pathogenic	0	0.0049
VWF	fs.del	NM_000552:exon28:c.4414delG:p.Asp1472ThrfsTer53	0.005447*	0.004367*⊥	NA	Likely Pathogenic	0	0.0016
VWF	nonsyn.	NM_000552:exon28:c.4414G>A:p.Asp1472Asn	0.002335*	0.004991⊥	0.003	VUS	0	0.0049
VWF	nonsyn.	NM_000552:exon28:c.4163A>G:p.Gln1388Arg	0.002335⊥	0.00156⊥	14.42	VUS	0.002335	0.00156
VWF	nonsyn.	NM_000552:exon28:c.3807T>A:p.Asp1269Glu	0.003113⊥	0.003743⊥	0.001	VUS	0.003113	0.0037
VWF	fs.ins	NM_000552:exon28:c.3805_3806insT:p.Asp1269ValfsTer 24	0.007782*	0.011853⊥	NA	Likely Pathogenic	0	0.011
VWF	nonsyn.	NM_000552:exon28:c.3806A>T:p.Asp1269Val	0.007782*	0.011853*⊥	7.382	VUS	0	0.0076
VWF	nonsyn.	NM_000552:exon28:c.3688G>A:p.Val1230Ile	0.000778⊥	0.000312⊥	13.77	VUS	0.000778	0
VWF	fs.ins	NM_000552:exon25:c.3358_3359insC:p.Trp1120fs	0.002335*	0.001248*	NA	Likely Pathogenic	0.000778	0.0012
VWF	nonsyn.	NM_000552:exon25:c.3286G>C:p.Asp1096His	0.000778⊥	0	29.3	VUS	0.000778	0
VWF	fs.ins	NM_000552:exon24:c.3157dupC:p.Gln1053ProfsTer9	0.000778*	0	NA	Pathogenic	0	0
VWF	nonsyn.	NM_000552:exon23:c.2981G>T:p.Gly994Val	0.006226*⊥	0	34	VUS	0.001556	0
VWF	nonsyn.	NM_000552:exon16:c.2182A>G:p.Met728Val	0.000778⊥	0.000312⊥	7.2	VUS	0.000778	0.0003
VWF	nonsyn.	NM_000552:exon16:c.2174A>G:p.His725Arg	0.000778*	0.0006*⊥	26.5	VUS	0	0.0003
VWF	nonsyn.	NM_000552:exon15:c.1916G>A:p.Arg639His	0.000778⊥	0	18.63	VUS	0.000778	0
VWF	fs.ins	NM_000552:exon15:c.1843dupT:p.Cys615LeufsTer35	0.000778*	0	NA	Pathogenic	0	0
VWF	splice	NM_000552:exon15:c.1730-1G>T	0.001556*	0	27	Pathogenic	0	0
VWF	splice	NM_000552:exon10:c.1110-1G>T	0.001556*⊥	0.000312⊥	26.5	Pathogenic	0.000778	0.0003
VWF	nonsyn.	NM_000552:exon8:c.960G>A:p.Met320Ile	0.000778⊥	0.000312⊥	21.4	VUS	0.000778	0.0003
VWF	fs.del	NM_000552:exon8:c.949delA: p.Ile317SerfsTer140	0.000778⊥	0.000312⊥	NA	Likely Pathogenic	0.000778	0.0003
VWF	nonsyn.	NM_000552:exon6:c.575C>T:p.Aal192Val	0.000778⊥	0.000312⊥	25.5	VUS	0.000778	0.0003
F7	nonsyn.	NM_019616:exon2:c.84A>C:p.Glu28Asp	0.000778⊥	0	1.285	Likely Benign	0.000778	0
F7	nonsyn.	NM_019616:exon2:c.220A>G:p.Arg74Gly	0.000778*	0.00156*⊥	23.7	Likely Pathogenic	0	0.0008
F7	nonsyn.	NM_001267554:exon3:c.205A>C:p.Asn69His	0.000778*	0.000312⊥	0.002	VUS	0	0.0003
F7	stopgain	NM_001267554:exon3:c.208G>T:p.Glu70Ter	0.000778*	0.000312⊥	35	Pathogenic	0	0.0003
F7	nonsyn.	NM_001267554:exon3:c.222T>G:p.Cys74Trp	0.000778⊥	0.000312⊥	23.4	VUS	0.000778	0.0003
F7	nonsyn.	NM_001267554:exon5:c.551T>C:p.Leu184Pro	0.000778⊥	0	24.5	Likely Pathogenic	0.000778	0
F7	stopgain	NM_001267554:exon6:c.1086G>A:p.Trp362Ter	0.000778⊥	0	40	Pathogenic	0.000778	0
F7	nonsyn.	NM_001267554:exon6:c.1106C>T:p.Ser369Leu	0.000778⊥	0	16.28	Likely Benign	0.000778	0

F7	nonsyn.	NM_001267554:exon6:c.1133G>T:p.Arg378Leu	0.001556⊥	0	23.6	VUS	0.001556	0
F10	nonsyn.	NM_000504:exon1:c.28C>T:p.Leu10Phe	0.000778⊥	0	13.59	VUS	0.000778	0
F10	nonsyn.	NM_000504:exon2:c.80G>A:p.Arg27His	0.002335⊥	0.000624⊥	6.846	VUS	0.002335	0.000624
F10	nonsyn.	NM_000504:exon2:c.84G>T:p.Arg28Ser	0.000778⊥	0	1.675	VUS	0.000778	0
F10	nonsyn.	NM_000504:exon2:c.89A>G:p.Gln30Arg	0.001556⊥	0	0.001	VUS	0.001556	0
F10	nonsyn.	NM_000504:exon2:c.107C>A:p.Ala36Glu	0.000778⊥	0.000624⊥	0.006	VUS	0.000778	0.000624
F10	nonsyn.	NM_000504:exon2:c.152G>A:p.Gly51Glu	0.003891⊥	0	23.6	VUS	0.003891	0
F10	fs.del	NM_001312674:exon5:c.378delC:p.Cys128ValfsTer95	0.002335⊥	0.000936⊥	NA	Pathogenic	0.002335	0
F10	nonsyn.	NM_001312674:exon6:c.697T>A:p.Cys233Ser	0.003113*⊥	0	24.7	VUS	0.001556	0
F10	nonsyn.	NM_001312674:exon7:c.1193G>A:p.Gly398Asp	0.001556⊥	0	24.4	VUS	0.001556	0
F10	nonsyn.	NM_001312674:exon7:c.1255G>C:p.Asp419His	0.000778*	0.000312⊥	12.7	VUS	NA	NA
HBA2	nonsyn.	NM_000517:exon2:c.199C>A:p.Leu67Met	0.000778⊥	0.000294⊥	23.2	VUS	NA	NA
HBA2	nonsyn.	NM_000517:exon2:c.200T>A:p.Leu67Gln	0.000778*	0.000294⊥	23.9	VUS	NA	NA
HBA2	nonsyn.	NM_000517:exon2:c.200T>G:p.Leu67Arg	0.000778*	0.000294⊥	24	VUS	NA	NA
HBA2	nonsyn.	NM_000517:exon2:c.203C>T:p.Thr68Ile	0.000778⊥	0.000294⊥	4.933	VUS	NA	NA
HBA2	fs.ins	NM_000517:exon2:c.210_211insT:p.Val71CysfsTer103	0.000778*	0.000294⊥	NA	Pathogenic	NA	NA
HBA2	nonsyn.	NM_000517:exon3:c.404C>T:p.Thr135Ile	0.046693⊥	0.001766⊥	13.84	VUS	NA	NA
F9	nonsyn.	NM_001313913:exon5:c.590C>A:p.Pro197Gln	0.000778⊥	0	19.19	VUS	NA	NA
G6PD	nonsyn.	NM_000402:exon12:c.1531C>G:p.Pro511Ala	0.000778*	0	7.925	VUS	NA	NA
G6PD	splice	NM_000402:exon6:c.734+2->G	0.000778*	0.000266⊥	NA	VUS	NA	NA
G6PD	nonsyn.	NM_000402:exon5:c.443A>C:p.Tyr148Ser	0.000778*	0	23.5	Likely Pathogenic	NA	NA
G6PD	nonsyn.	NM_000402:exon4:c.325G>C:p.Asp109His	0.000778*	0.000266*^	23.5	Likely Pathogenic	NA	NA
G6PD	nonsyn.	NM_000402:exon3:c.242C>T:p.Thr81Ile	0.000778⊥	0	27	VUS	NA	NA
F8	nonsyn.	NM_019863:exon4:c.429G>T:p.Glu143Asp	0.000778⊥	0.000294⊥	24.1	VUS	NA	NA
F8	nonsyn.	NM_019863:exon3:c.304G>T:p.Ala102Ser	0.000778⊥	0.000294⊥	33	VUS	NA	NA
F8	fs.del	NM_019863:exon3:c.273delA:p.Lys91fs	0.001556*	0	NA	Likely Pathogenic	NA	NA
F8	nonsyn.	NM_000132:exon22:c.6322G>C:p.Ala2108Pro	0.001556*	0	27.4	VUS	NA	NA
F8	nonsyn.	NM_000132:exon18:c.5863C>G:p.Gln1955Glu	0.000778*	0	9.465	VUS	NA	NA
F8	splice	NM_000132:exon18:c.5816-2A>G	0.000778*	0	23.1	Likely Pathogenic	NA	NA
F8	nonsyn.	NM_000132:exon17:c.5815G>C:p.Ala1939Pro	0.003113*⊥	0	27.9	VUS	NA	NA
F8	nonsyn.	NM_000132:exon15:c.5267A>T:p.Glu1756Val	0.003113*⊥	0	32	VUS	NA	NA
F8	nonsyn.	NM_000132:exon14:c.5005A>G:p.Ile1669Val	0.000778⊥	0	0.014	VUS	NA	NA
F8	fs.del	NM_000132:exon14:c.3780delC:p.Asp1260fs	0.000778*	0.004709*^	NA	Likely Pathogenic	NA	NA
F8	nonsyn.	NM_000132:exon14:c.3744A>T:p.Leu1248Phe	0.000778*	0	19.45	VUS	NA	NA

F8	stopgain	NM_000132:exon14:c.3266C>A:p.Ser1089Ter	0.000778*	0	35	Pathogenic	NA	NA
F8	nonsyn.	NM_000132:exon14:c.3144G>T:p.Trp1048Cys	0.000778⊥	0.000294⊥	25.1	VUS	NA	NA
F8	nonsyn.	NM_000132:exon14:c.2776C>T:p.Pro926Ser	0.001556⊥	0	0.002	VUS	NA	NA
F8	nonsyn.	NM_000132:exon14:c.2573C>T:p.Pro858Leu	0.000778⊥	0	10.76	VUS	NA	NA
F8	nonsyn.	NM_000132:exon13:c.1969T>C:p.Tyr657His	0.001556⊥	0.001177*^	8.426	VUS	NA	NA
F8	nonsyn.	NM_000132:exon11:c.1724A>C:p.Lys575Thr	0.000778⊥	0.000294⊥	26.6	VUS	NA	NA
F8	fs.del	NM_000132:exon4:c.494delC:.Pro165GlnfsTer20	0.002335*	0	NA	Pathogenic	NA	NA
F8	fs.ins	NM_000132:exon2:c.255dupA:p.Pro86ThrfsTer13	0.000778*	0	NA	Pathogenic	NA	NA
F8	fs.del	NM_000132:exon2:c.230delT:p.Phe78SerfsTer14	0.001556*	0.001471⊥	NA	Pathogenic	NA	NA

⊥ heterozygous, *homozygous/hemizygous males (X-chromosome genes) , *⊥ heterozygous and homozygous/ hemizygous males (X-chromosome genes). ^ Recorded as hemizygous in at least one male individual.

Nonsyn: nonsynonymous. fs.Ins: Frameshift insertion. fs.del: Frameshift deletion. P-cohort: Primary cohort. R-cohort: Replication cohort. (value= Case number/Total Cases).

Supplementary Table 5 Nonsynonymous Polymorphisms identified in our samples

Gene	Exon/Base and A.A change	P-Cohort	R-Cohort	dbSNP	CADD_phred
F5	NM_000130:exon23:c.6323G>A:p.Arg2108His	0.000778⊥	0.000437⊥	rs751124066	27.6
F5	NM_000130:exon19:c.5729C>T:p.Pro1910Leu	0.000778⊥	0.000873⊥	rs200805004	33
F5	NM_000130:exon15:c.5059G>A:p.Glu1687Lys	0.000778⊥	0	rs201556325	34
F5	NM_000130:exon14:c.4891C>T:p.Arg1631Cys	0.000778⊥	0.000655⊥	rs771962686	34
F5	NM_000130:exon13:c.4568T>C:p.Ile1523Thr	0.000778⊥	0.000218⊥	rs187520391	18.93
F5	NM_000130:exon13:c.4414T>G:p.Ser1472Ala	0.000778	0	rs749081272	0.018
F5	NM_000130:exon13:c.4405T>C:p.Ser1469Pro	0.000778⊥	0.001092	rs144262027	20.2
F5	NM_000130:exon13:c.4000T>C:p.Phe1334Leu	0.016342*⊥	0.00393*⊥	rs147741798	0.023
F5	NM_000130:exon13:c.3784C>T:p.Leu1262Phe	0.003113⊥	0.001965⊥	rs757445087	0.23
F5	NM_000130:exon13:c.3679G>C:p.Gly1227Arg	0.001556*⊥	0.000218⊥	rs549903844	18.46
F5	NM_000130:exon13:c.3659G>C:p.Arg1220Thr	0.042023⊥	0.006987⊥	rs769422860	0.001
F5	NM_000130:exon13:c.3653T>G:p.Ile1218Ser	0.017121⊥	0.003712⊥	rs762371932	0.076
F5	NM_000130:exon13:c.3311C>A:p.Ser1104Tyr	0.001556⊥	0.000437⊥	rs188882337	0.502
F5	NM_000130:exon13:c.3257T>C:p.Leu1086Ser	0.000778⊥	0.000873⊥	rs116407347	9.814
F5	NM_000130:exon13:c.3211C>T:p.His1071tyr	0.000778⊥	0.001092	rs146408488	14.55
F5	NM_000130:exon13:c.3149C>T:p.Pro1050Leu	0.000778⊥	0	rs761897827	23.3
F5	NM_000130:exon13:c.3092A>G:p.Lys1031Arg	0.003113⊥	0.001965⊥	rs750884507	13.07
F5	NM_000130:exon13:c.2756C>T:p.Ser919Phe	0.000778⊥	0.000218⊥	rs774750553	23.7
F5	NM_000130:exon13:c.2119C>T:p.Arg707Trp	0.000778⊥	0	rs139222212	25.8
F5	NM_000130:exon10:c.1531A>G:p.Ile511Val	0.000778⊥	0	rs759369619	0.031

F5	NM_000130:exon7:c.1106C>T:p.Ala369Val	0.000778⊥	0	rs200934105	22.9
F5	NM_000130:exon7:c.1033C>T:p.Arg345Trp	0.002335⊥	0.000437⊥	rs746260106	33
F5	NM_000130:exon4:c.566C>T:p.Pro189Leu	0.002335⊥	0.00131*⊥	rs141800405	26
F5	NM_000130:exon4:c.511A>G:p.Ile171Val	0.000778⊥	0.000218⊥	rs1003110107	22.7
F5	NM_000130:exon1:c.112G>A:p.Ala38Thr	0.001556⊥	0.00131⊥*	rs184663825	27.5
F13B	NM_001994:exon7:c.1060T>C:p.Tyr354His	0.000778⊥	0.000936⊥	rs376295989	19.17
FGB	NM_001184741:exon1:c.97G>A:p.Val33Ile	0.000778⊥	0	rs562142566	0.002
FGB	NM_001184741:exon5:c.585G>A:p.Met195Ile	0.000778⊥	0.000312⊥	rs201536638	23.3
FGB	NM_001184741:exon7:c.1063G>A:p.Gly355Ser	0.009339*⊥	0.014036⊥	rs141881199	23.3
FGA	NM_000508:exon6:c.2089G>A:p.Gly697Ser	0.001556⊥	0	rs771023837	29.7
FGA	NM_000508:exon6:c.1961G>A:p.Gly654Glu	0.000778⊥	0	rs140959479	25.2
FGA	NM_000508:exon5:c.1570T>A:p.Ser524Thr	0.000778⊥	0.000195⊥	rs1009519911	2.048
FGA	NM_000508:exon5:c.1529G>A:p.Arg510His	0.007782*⊥	0.005061*⊥	rs138137585	7.406
FGA	NM_000508:exon5:c.1055C>A:p.Pro352His	0.000778⊥	0	rs373154873	22.7
FGA	NM_000508:exon5:c.1025G>A:p.G342E	0.004669⊥	0.003504*⊥	rs774664670	8.039
FGA	NM_000508:exon5:c.626C>A:p.Ala209Asp	0.000778⊥	0	rs754490462	25.4
FGA	NM_000508:exon5:c.514G>A:p.Asp172Asn	0.006226⊥	0.002336⊥	rs748106542	30
FGA	NM_000508:exon4:c.389T>C:p.Val130Ala	0.000778⊥	0	rs565128682	22.8
FGG	NM_000509:exon7:c.671T>G:p.Leu224Arg	0.000778⊥	0	rs573927803	23.7
F11	NM_000128:exon5:c.430G>A:p.Val144Ile	0.002335⊥	0.003743⊥	rs991720364	0.002
F11	NM_000128:exon6:c.536C>T:p.Thr179Met	0.000778⊥	0	rs766929913	0.002
F11	NM_000128:exon9:c.877T>G:p.Ser293Ala	0.000778⊥	0	rs758385654	0.004

F11	NM_000128:exon10:c.1128G>A:p.Met376Ile	0.000778⊥	0	rs763964150	23.8
F11	NM_000128:exon12:c.1328G>A:p.Arg443His	0.000778⊥	0.000312⊥	rs373212439	23.4
F13A1	NM_000129:exon15:c.2130G>A:p.Met710Ile	0.000778⊥	0.000312⊥	rs140537838	24.4
F13A1	NM_000129:exon14:c.1912C>T:p.Arg638Cys	0.000778⊥	0	rs200803360	26.5
F13A1	NM_000129:exon8:c.998C>T:p.Ala333Val	0.000778⊥	0.000312⊥	rs759294043	32
F13A1	NM_000129:exon4:c.508G>A:p.Val170Ile	0.000778⊥	0	rs140712764	0.172
F13A1	NM_000129:exon4:c.476G>A:p.Arg159His	0.000778⊥	0.000312⊥	rs747218826	29.5
F2	NM_000506:exon2:c.118C>T:p.Arg40Trp	0.002335*⊥	0.000218⊥	rs1013222460	28.5
F2	NM_000506:exon7:c.671G>A:p.Arg224His	0.002335⊥	0.001965*⊥	rs200525645	11.89
F2	NM_000506:exon7:c.859G>A:p.Asp287Asn	0.000778⊥	0.000218⊥	rs777315807	22.6
F2	NM_000506:exon12:c.1542C>A:p.Asn514lys	0.007782⊥	0.00393*⊥	rs199772906	11.23
F2	NM_000506:exon12:c.1628G>A:p.Asg543His	0.000778⊥	0.000437⊥	rs143064939	24.8
HBB	NM_000518:exon3:c.380T>C:p.Val127Ala	0.000778⊥	0.000312⊥	rs33925391	4.985
VWF	NM_000552:exon42:c.7126C>T:p.Pro2376Ser	0.000778⊥	0.000312⊥	rs775107211	24.6
VWF	NM_000552:exon40:c.6931C>T:p.Arg2311Cys	0.000778⊥	0	rs150725355	24.9
VWF	NM_000552:exon39:c.6878C>T:p.Thr2293Met	0.002335⊥	0.002183⊥	rs142921275	7.506
VWF	NM_000552:exon37:c.6479A>G:p.Tyr2160Cys	0.000778⊥	0.000312⊥	rs779764302	23.5
VWF	NM_000552:exon34:c.5739G>C:p.Lys1913Asn	0.000778⊥	0.000312⊥	rs753735116	17.55
VWF	NM_000552:exon32:c.5542G>A:p.Val1848Met	0.000778⊥	0.000312⊥	rs376993019	19.83
VWF	NM_000552:exon32:c.5533G>A:p.Asp1845Asn	0.000778⊥	0	rs201548925	1.088
VWF	NM_000552:exon31:c.5384C>T:p.Ala1795Val	0.000778⊥	0.000312⊥	rs146729537	14
VWF	NM_000552:exon31:c.5369C>T:p.Pro1790Leu	0.001556⊥	0.000624⊥	rs551649729	25

VWF	NM_000552:exon30:c.5177G>A:p.Arg1726His	0.000778$^{\perp}$	0.000312$^{\perp}$	rs147313320	20.3
VWF	NM_000552:exon28:c.4967C>T:p.Thr1656Met	0.000778$^{\perp}$	0	rs773544469	10.45
VWF	NM_000552:exon28:c.4732A>G:p.Thr1578Ala	0.000778$^{\perp}$	0	rs913807168	22.8
VWF	NM_000552:exon28:c.4201G>A:p.Val1401Ile	0.000778$^{\perp}$	0.000312$^{\perp}$	rs536484748	14.76
VWF	NM_000552:exon28:c.4007G>A:p.Arg1336Gln	0.000778$^{\perp}$	0	rs886049741	23.4
VWF	NM_000552:exon25:c.3328G>A:p.Val1110Met	0.007004*$^{\perp}$	0.008422*$^{\perp}$	rs763577959	23
VWF	NM_000552:exon21:c.2717G>A:p.Arg906Gln	0.000778$^{\perp}$	0.000312$^{\perp}$	rs542975251	24.1
VWF	NM_000552:exon15:c.1753G>A:p.Ala585Tht	0.000778$^{\perp}$	0.000312$^{\perp}$	rs141777100	24.2
VWF	NM_000552:exon13:c.1516G>A:p.Gly506Arg	0.000778$^{\perp}$	0	rs201456647	35
VWF	NM_000552:exon9:c.1027G>T:p.Val343Leu	0.000778$^{\perp}$	0.003743$^{\perp}$	rs746575545	24.2
VWF	NM_000552:exon5:c.430G>A:p.Gly144Ser	0.000778$^{\perp}$	0	rs781180895	27.4
VWF	NM_000552:exon3:c.126C>G:p.Phe42Leu	0.002335$^{\perp}$	0.001248$^{\perp}$	rs200710351	12.33
F7	NM_000131:exon2:c.89G>A:p.Gly30Glu	0.000778$^{\perp}$	0.000312$^{\perp}$	rs760606639	0.004
F7	NM_000131:exon2:c.97C>T:p.Arg33Trp	0.003891$^{\perp}$	0.002495$^{\perp}$	rs764010176	14.61
F7	NM_001267554:exon4:c.400G>A:p.Val134Met	0.001556$^{\perp}$	0.000312$^{\perp}$	rs139309572	9.636
F7	NM_001267554:exon6:c.1095G>C:p.Lys365Asp	0.000778$^{\perp}$	0	rs769953249	4.56
F7	NM_001267554:exon6:c.1115G>A:p.Arg372His	0.000778$^{\perp}$	0	rs767892850	0.834
F10;	NM_000504:exon2:c.71T>C:p.Lys24Pro	0.002335$^{\perp}$	0.001248$^{\perp}$	rs372382600	21.9
F10	NM_000504:exon5:c.454G>A:p.Ala152Thr	0.000778$^{\perp}$	0.00156*$^{\perp}$	rs3211772	23.6
F10	NM_001312675:exon8:c.899C>T:p.Thr300Met	0.000778$^{\perp}$	0	rs146421172	10.75
F10	NM_001312675:exon8:c.911G>A:p.Trp304Ter	0.003891*$^{\perp}$	0.000624$^{\perp}$	rs140955427	4.385
F10	NM_001312675:exon8:c.934C>T:p.Gln312Ter	0.000778$^{\perp}$	0	rs536969626	5.279

F10	NM_001312674:exon7:c.841G>A:p.Val281Met	0.000778[⊥]	0	rs373791924	21.3
HBA2	NM_000517:exon2:c.197C>T:p.Ala66Val	0.000778[⊥]	0.000294[⊥]	rs281864851	24.4
F9	NM_001313913:exon7:c.1267A>C:p.Thr423Pro	0.000778[⊥]	0.000883[⊥]	rs4149751	23.2
G6PD	NM_000402:exon9:c.1079G>A:p.Arg360His	0.007782*[⊥]	0.007441*[⊥]	rs868950643	7.666
G6PD	NM_000402:exon6:c.649A>G:p.Ile217Val	0.000778[⊥]	0	rs782366444	22.7
G6PD	NM_000402:exon5:c.407C>T:p.Ser136Phe	0.000778*	0	rs267606835	9.177
G6PD	NM_000402:exon4:c.354C>G:p.Phe118Leu	0.000778[⊥]	0	rs781794862	2.678
F8	NM_000132:exon15:c.5299T>C:p.Tyr1767His	0.000778*	0.002649*[⊥]	rs781791811	14.52
F8	NM_000132:exon14:c.4978C>T:p.Pro1660Ser	0.000778*	0.000294*[⊥]	rs200274569	0.002
F8	NM_000132:exon14:c.3980C>T:p.Thr1327Met	0.003113[⊥]	0.003531*[⊥]	rs200520711	3.104
F8	NM_000132:exon14:c.3376A>T:p.Arg1126Trp	0.000778*	0.000294*[⊥]	rs199630813	16.01
F8	NM_000132:exon11:c.1546C>T:p.His516Tyr	0.000778[⊥]	0	rs782659883	24
F8	NM_000132:exon9:c.1286A>G:p.Gln429Arg	0.003891*[⊥]	0.002649*[⊥]	rs868946748	0.117

[⊥] heterozygous, *homozygous , *[⊥] heterozgyous and homozygous/hemizygous

Supplementary Table 6 Frequency of heterozygous carriers of HGMD and novel variants in the AR target genes

	F2	***F5***	***F7***	***F10***	***F11***	***F13A***	***F13B***	***FGA***	***FGB***	***FGG***	***HBB***	***VWF***
**P-Cohort**	0.007	0.012	0.010	0.016	0.002	0.005	0.006	0.007	0.000	0.004	0.010	0.117
**R-Cohort**	0.001	0.005	0.005	0.002	0.003	0.001	0.001	0.003	0.000	0.000	0.003	0.084

Carrier frequency is calculated for pathogenic, likely pathogenic and VUS variants. Carrier Frequency= Sample Count/Total Count.

Supplementary Table 7 Percentage of heterozygous carriers for HGMD and novel variants in P-Cohort

	**P-Cohort**		**R-Cohort**	
**Gene**	**HGMD**	**Novel**	**HGMD**	**Novel**
F2	0.08%	0.62%	0.02%	0.07%
F5	0.00%	1.17%	0.00%	0.52%
F7	0.62%	0.39%	0.22%	0.25%
F10	0.08%	1.56%	0.00%	0.25%
F11	0.16%	0.08%	0.00%	0.31%
F13A1	0.23%	0.31%	0.06%	0.00%
F13B	0.00%	0.62%	0.00%	0.12%
FGA	0.00%	0.70%	0.00%	0.27%
FGB	0.00%	0.00%	0.00%	0.00%
FGG	0.08%	0.31%	0.00%	0.03%
HBB	0.93%	0.08%	0.25%	0.03%
VWF	0.86%	10.82%	0.06%	8.30%

Carrier frequency is calculated for pathogenic, likely pathogenic and VUS variants/gene. heterozygous Carrier Frequency= (Sample Count/Total Count)*100

Supplementary Table 8 List of Genes selected in this study

Gene	RefSeq/ENSG Accession	OMIM Accession	Disease	Mode of inheritance	Number of HGMD mutations
F10	NM_001312674/ ENSG00000126218	613872	Factor X deficiency	AR	135
F11	NM_000128/ ENSG00000088926	264900	Factor XI deficiency	AR/AD	236
F13A1	NM_000129/ ENSG00000124491	134570	Factor XIII deficiency/Venous thrombosis, protection against	AR/AD	146
F13B	NM_001994/ ENSG00000143278	134580	Factor XIIIB deficiency	AR	
F2	NM_000506/ ENSG00000180210	176930	Prothrombin deficiency(Dysprothrombinemia)/Hypoprothrombinemia/Thrombophilia due to thrombin defect	AR/AD/AD	65
F5	NM_000130/ ENSG00000198734	612309	Factor V deficiency/Thrombophilia due to activated protein C resistance/Thrombophilia, susceptibility to, due to factor V Leiden	AR/AD/AD	159
F7	NM_001267554/ ENSG00000057593	613878	Factor VII deficiency	AR	290
F8	NM_019863/ ENSG00000185010	300841	Hemophilia A (factor VIII deficiency)	XLR	2928
F9	NM_000133/ ENSG00000101981	300746	Hemophilia B (factorIX deficiency)/Warfarin sensitivity	XLD/AD	1218
FGA	NM_000508/ ENSG00000171560	134820	Afibrinogenemia, congenital/Amyloidosis, familial visceral	AR/AD	129
FGB	NM_005141/ ENSG00000171564	134830	Afibrinogenemia, congenital/Hypofibrinogenemia, congenital	AR	178
FGG	NM_000509/ ENSG00000171557	134850	Afibrinogenemia, congenital/Hypofibrinogenemia, congenital	AR	114
G6PD	NM_000402/ ENSG00000160211	305900	Hemolytic anemia, G6PD deficient (favism)	XLD	234
HBA1	NM_000558/ENSG 00000206172	141800	Heinz body anemias, alpha-	AD	147
HBA2	NM_000517/ ENSG00000188536	141850	Heinz body anemia	AD	231
HBB	NM_000518/ ENSG00000244734	141900	Thalassemia, beta/Delta-beta thalassemia	AR/AD	790
VWF	NM_000552/ ENSG00000110799	613160	Von Willebrand disease	AD/AR	1006

***REfSeq: Reference Sequence accession number. ENSG: Ensembl accession number. OMIM: Online Mendelian Inheritance in Man. (Access dates Dec 2020).**

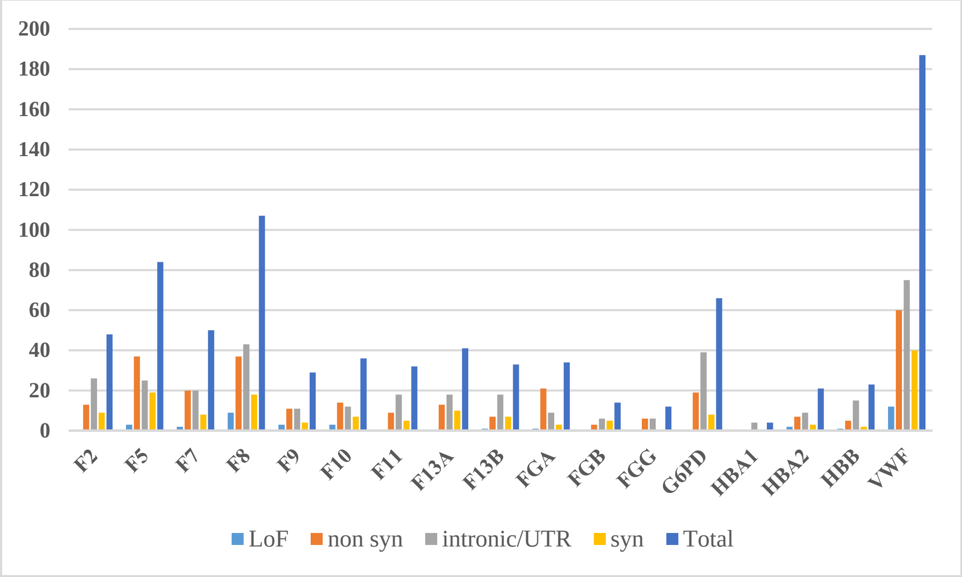

Supplementary Figure 1 Breakdown of types variants identified in the targeted genes. LoF: Loss of function mutations. Non syn: Nonsynonymous variants, Syn: Synonymous variants. (Figure is generated from Supplementary Table 2)
