# Supplementary material for: Molecular classification of blood and bleeding disorder genes
Source: NPJ Genom Med. 2021 Jul 16;6:62. doi: 10.1038/s41525-021-00228-2 (PMC8285395; doi:10.1038/s41525-021-00228-2)
Supplement: Supplementary file 2 — Reporting Summary [file 41525_2021_228_MOESM2_ESM.pdf]

nature research

Corresponding author(s): NPJGENMED-00897_N.A.T

Last updated by author(s): Jun 13, 2021

Reporting Summary

Nature Research wishes to improve the reproducibility of the work that we publish. This form provides structure for consistency and transparency in reporting. For further information on Nature Research policies, see our Editorial Policies and the Editorial Policy Checklist.

Statistics

For all statistical analyses, confirm that the following items are present in the figure legend, table legend, main text, or Methods section.

n/a	Confirmed	
☐	☒	The exact sample size (n) for each experimental group/condition, given as a discrete number and unit of measurement
☐	☒	A statement on whether measurements were taken from distinct samples or whether the same sample was measured repeatedly
☒	☐	The statistical test(s) used AND whether they are one- or two-sided<br>*Only common tests should be described solely by name; describe more complex techniques in the Methods section.*
☐	☒	A description of all covariates tested
☒	☐	A description of any assumptions or corrections, such as tests of normality and adjustment for multiple comparisons
☒	☐	A full description of the statistical parameters including central tendency (e.g. means) or other basic estimates (e.g. regression coefficient) AND variation (e.g. standard deviation) or associated estimates of uncertainty (e.g. confidence intervals)
☒	☐	For null hypothesis testing, the test statistic (e.g. F, t, r) with confidence intervals, effect sizes, degrees of freedom and P value noted<br>*Give P values as exact values whenever suitable.*
☒	☐	For Bayesian analysis, information on the choice of priors and Markov chain Monte Carlo settings
☒	☐	For hierarchical and complex designs, identification of the appropriate level for tests and full reporting of outcomes
☒	☐	Estimates of effect sizes (e.g. Cohen's d, Pearson's r), indicating how they were calculated

Our web collection on statistics for biologists contains articles on many of the points above.

Software and code

Policy information about availability of computer code

Data collection	NA
Data analysis	1- Alignment/Variant Calling: Ion Torrent - Torrent Suite (TS version 5.12) https://github.com/iontorrent/TS<br>2- Annotation: Annovar System (https://annovar.openbioinformatics.org/en/latest/) [v 20210123]

For manuscripts utilizing custom algorithms or software that are central to the research but not yet described in published literature, software must be made available to editors and reviewers. We strongly encourage code deposition in a community repository (e.g. GitHub). See the Nature Research guidelines for submitting code & software for further information.

Data

Policy information about availability of data

All manuscripts must include a data availability statement. This statement should provide the following information, where applicable:

- Accession codes, unique identifiers, or web links for publicly available datasets
- A list of figures that have associated raw data
- A description of any restrictions on data availability

All data generated or analyzed during this study are included in this published article (and its supplementary information files). The novel mutations have been also deposited in Clinvar NCBI site (Accession numbersSCV001622445 - SCV001622583). Furthermore, the variant files of this study that can be neither deposited to Clinvar nor formatted as supplementary tables have been made available via figshare DOI (https://doi.org/10.6084/m9.figshare.14785911) as per journal policy. Additional data will be available upon request for research purposes.

Field-specific reporting

Please select the one below that is the best fit for your research. If you are not sure, read the appropriate sections before making your selection.

☒ Life sciences ☐ Behavioural & social sciences ☐ Ecological, evolutionary & environmental sciences

For a reference copy of the document with all sections, see nature.com/documents/nr-reporting-summary-flat.pdf

Life sciences study design

All studies must disclose on these points even when the disclosure is negative.

Sample size	Two cohorts used in this study; the primary anonymous cohort (P-cohort) of 1285 cases and family members with a possibility hematological disease, mainly bleeding disorder analyzed using targeted gene sequencing. The secondary (replication cohort; R-cohort)of a maximum of 5000 ethnically matched exomes from cases not related or diagnosed with bleeding or blood disorders and were sequenced as part of SHGP
Data exclusions	Seventeen genes were selected for analysis (13 of which are linked to different bleeding disorders and 4 related to common blood disorders). Genes associated with Glanzmann Thrombasthenia, a rare genetic bleeding disorder, were excluded since they were studied and evaluated in an earlier study
Replication	The secondary (replication cohort, R-cohort) of a maximum of 5000 ethnically matched exomes from cases not related or diagnosed with bleeding or blood disorders and were used to determine the frequency of variants identified in the P-cohort in the main population.
Randomization	R-cohort was randomly selected
Blinding	NA

Reporting for specific materials, systems and methods

We require information from authors about some types of materials, experimental systems and methods used in many studies. Here, indicate whether each material, system or method listed is relevant to your study. If you are not sure if a list item applies to your research, read the appropriate section before selecting a response.

Materials & experimental systems

n/a	Involved in the study	
☒	☐	Antibodies
☒	☐	Eukaryotic cell lines
☒	☐	Palaeontology and archaeology
☒	☐	Animals and other organisms
☐	☒	Human research participants
☒	☐	Clinical data
☒	☐	Dual use research of concern

Methods

n/a	Involved in the study	
☒	☐	ChIP-seq
☒	☐	Flow cytometry
☒	☐	MRI-based neuroimaging

Human research participants

Policy information about studies involving human research participants

Population characteristics	The investigate cohort (P- cohort) are of samples recruited for blood and bleeding disorder studies. The replication cohort (R-cohort) are randomly local exomes. No age or gender selection.<br>Samples are from Saudi Arabia (Arab ethnic group)
Recruitment	Samples were recruited for approved NGS studies.
Ethics oversight	All samples at the SHGP are consented with ethical approval that covers use of anonymized data for analysis. The specified samples used for this study (P-cohort) are part of an approved project (RAC#2130036) at King Faisal Specialist Hospital and Research Center (KFSH&RC).

Note that full information on the approval of the study protocol must also be provided in the manuscript.
